# Blood absolute lymphocyte count and trajectory are important in understanding severe COVID-19

**DOI:** 10.1186/s12879-024-10428-7

**Published:** 2025-01-15

**Authors:** Catharine I. Paules, Jacqueline A. Nordwall, Kathryn Shaw-Saliba, Judith A. Aberg, Edward M. Gardner, Anna L. Goodman, N. Kumarasamy, Shikha Vasudeva, David M. Vock, Crystal M. North, Jens Lundgren, Neil R. Aggarwal, for the STRIVE Network and TICO Trial Study Group

**Affiliations:** 1 Division of Infectious Diseases, Penn State Health Milton S. Hershey Medical Center, 500 University Drive, Hershey, PA 17033 USA; 2 Division of Biostatistics and Health Data Science, School of Public Health, University of Minnesota, Minneapolis, MN USA; 3 Division of Clinical Research, National Institute of Allergy and Infectious Diseases, National Institutes of Health, Bethesda, MD USA; 4 Department of Medicine, Icahn School of Medicine at Mount Sinai, New York, NY USA; 5 Denver Health and Hospital, Authority, Denver, CO USA; 6 MRC Clinical Trials Unit at University College London and CIDR, King’s College London and Guy’s and St. Thomas’ NHS Foundation Trust, London, UK; 7 VHS Infectious Diseases Medical Centre, CART Clinical Research Site, Voluntary Health Services, Chennai, India; 8 Division of Infectious Diseases, VA Medical Center, Salem, VA USA; 9 Division of Pulmonary and Critical Care Medicine, Massachusetts General Hospital, Boston, MA USA; 10 Harvard Medical School, Boston, MA USA; 11 CHIP Center of Excellence for Health, Immunity, and Infections, Department of Infectious Diseases, University of Copenhagen, Righospitalet, Copenhagen, Denmark; 12 Division of Pulmonary Sciences and Critical Care Medicine, University of Colorado School of Medicine, Aurora, CO USA

**Keywords:** Lymphocytes, SARS-CoV-2, Precision medicine

## Abstract

**Background:**

Low blood absolute lymphocyte count (ALC) may predict severe COVID-19 outcomes. Knowledge gaps remain regarding the relationship of ALC trajectory with clinical outcomes and factors associated with lymphopenia.

**Methods:**

Our post hoc analysis of the Therapeutics for Inpatients with COVID-19 platform trial utilized proportional hazards models to assess relationships between Day (D) 0 lymphopenia (ALC < 0.9 cells/uL), D0 severe lymphopenia (ALC < 0.5 cells/uL) or lymphopenia trajectory between D0 and D5 with mortality and secondary infections, and with sustained recovery using Fine-Gray models. Logistic regression was used to assess relationships between clinical variables and D0 lymphopenia or lymphopenia trajectory.

**Results:**

D0 lymphopenia (1426/2579) and severe lymphopenia (636/2579) were associated with increased mortality (aHR 1.48; 1.08, 2.05, *p* = 0.016 and aHR 1.60; 1.20, 2.14, *p* = 0.001) and decreased recovery (aRRR 0.90; 0.82, 0.99, *p* = 0.033 and aRRR 0.78; 0.70, 0.87, *p* < 0.001 respectively). Trial participants with persistent D5 lymphopenia had increased mortality, and increased secondary infections, and participants with persistent or new lymphopenia had impaired recovery, as compared to participants with no lymphopenia. Persistent and new lymphopenia were associated with older age, male sex; prior immunosuppression, heart failure, aspirin use, and normal body mass index; biomarkers of organ damage (renal and lung), and ineffective immune response (elevated IL-6 and viral nucleocapsid antigen levels). Similar results were observed with severe lymphopenia.

**Conclusions:**

Lymphopenia was predictive of severe COVID-19 outcomes, particularly when persistent or new during hospitalization. A better understanding of the underlying risk factors for lymphopenia will help illuminate disease pathogenesis and guide management strategies.

**Supplementary Information:**

The online version contains supplementary material available at 10.1186/s12879-024-10428-7.

## Introduction

Variability in immune function influences clinical presentation and prognosis for infectious diseases such as community acquired pneumonia (CAP) [1], bacterial sepsis [2], and influenza [3]. In certain patients, a phenotype of immune dysregulation occurs in which individuals cannot reduce pathogen burden or limit deleterious inflammation. Identifying such patients is crucial for clinical management, elucidating disease pathogenesis, designing robust clinical trials, and targeting therapeutic interventions precisely. In COVID-19, a number of biomarkers have been assessed with the goal of identifying patients at highest risk for severe outcomes.

Blood absolute lymphocyte count (ALC) is of interest as a prognostic biomarker for hospitalized patients with infection as it is easily measured in clinical care and has been associated with mortality in studies of CAP and sepsis, among others [4–6]. Notably, over half of hospitalized COVID-19 patients present with lymphopenia, and ALC values between 400 and 800 lymphocytes/L have been associated with intensive care unit admission, acute respiratory distress syndrome, and death [7–12]. This suggests an important relationship between COVID-19 pathogenesis and the lymphocyte pool that can be measured by clinicians at bedside. While limited data suggest that persistent lymphopenia is associated with worse outcomes in critically ill patients [13, 14] including those with COVID-19 [15, 16], knowledge gaps remain regarding the relationship of ALC trajectory with clinical outcomes. In addition, few studies have assessed the demographic, clinical, and laboratory characteristics associated with ALC trends [16]. To address these knowledge gaps, we conducted a post hoc analysis of data from the Therapeutics for Inpatients with COVID-19 (TICO) platform trials, building on a prior study that evaluated the association of baseline variables with mortality [12].

## Methods

### Data set

The TICO platform trial enrolled 2753 hospitalized participants aged 18 and older from August 2020 through November 2021 across 114 international sites in the Institutional Review Board approved trial (NCT04501978). 2625 (95.4%) participants were randomly assigned to receive one of five antiviral products (bamlanivimab [17], sotrovimab [18], amubarvimab–romlusevimab [18], tixagevimab–cilgavimab [19], and ensovibep [20] or matched placebo and received all or part of the assigned study product [modified intention to treat (mITT) population]. The primary outcome was time to sustained clinical recovery up to day 90, defined as the time from randomization to return to prior residence for 14 consecutive days and mortality by day 90 was a key secondary outcome. We describe trial platform eligibility criteria in the supplement. Only participants with ALC measured in the 24 h prior to randomization were included in this analysis.

### Clinical data

Demographic, clinical, and COVID-19 related characteristics were collected on case report forms (see Supplement). Oxygen requirement was assessed daily from day 0 (the day of randomization; D0) until day 5 (D5) and was defined as the maximum requirement needed each day. Concomitant medications were assessed for the 24 h prior to randomization and the 24 h prior to D5 except for remdesivir where the first and last dates of administration were recorded. Secondary infections were collected through day 90 and were defined as “intercurrent at least probable documented serious disease caused by an infection other than SARS-CoV-2, requiring antimicrobial administration and care at an acute-care hospital.”

### Laboratory measurements

Estimated glomerular filtration rate (eGFR), creatinine, and ALC were measured at each study site’s clinical laboratory on D0 and D5. A panel of biomarkers were measured centrally using banked plasma samples that were collected on days 0, 1, 3, or 5 and included quantitative plasma SARS-Cov-2 Nucleocapsid antigen (N-Ag) (Quanterix), SARS-CoV-2 viral RNA from mid-turbinate swab, anti-spike pseudo neutralizing antibody (GenScript), anti-Nucleocapsid antibody (Bio-Rad), Interleukin 6 (IL-6), C-Reactive Protein (CRP), and D-dimer (see supplement).

### Statistical analysis: relationship between lymphopenia and participant outcome

We defined lymphopenia as ALC < 0.9 × 10^9^/L and severe lymphopenia as ALC < 0.56 × 10^9^/L, which was the lowest quartile of D0 values in our population.

We examined associations between D0 lymphopenia and severe lymphopenia with mortality and secondary infections through day 90 using multivariable Cox proportional hazards models. Adjusted recovery rate ratios (RRRs), also known as subdistribution hazard ratios, for sustained clinical recovery were computed using adjusted Fine–Gray models, treating death as a competing risk. All models were adjusted for age, sex, race/ethnicity, residence, geographical region, infection period, baseline pulmonary status, and D0 biomarkers including plasma N-Ag, SARS-CoV-2 viral load, eGFR, CRP, IL-6, and D-dimer. Follow-up began at randomization and continued until the event of interest, Day 90 (D90), or the participant was lost to follow-up or withdrew consent.

We then evaluated the relationship between lymphopenia trajectory and these outcomes. We divided participants who were alive and had available D5 ALC into four groups based on each individual’s lymphocyte trajectory; (i) not lymphopenic at either timepoint (no lymphopenia), (ii) lymphopenic at D0 but not at D5 (resolved lymphopenia), (iii) not lymphopenic at D0 but lymphopenic at D5 (new lymphopenia), (iv) lymphopenic at D0 and D5 (persistent lymphopenia). Cumulative incidence curves for mortality and sustained recovery were estimated using Kaplan-Meier and Aalen-Johansen curves, respectively. We then examined associations between each group with mortality, secondary infections, and sustained clinical recovery using the adjusted models described above. In the Cox proportional hazard and Fine-Gray models including D5 lymphopenia, follow-up began at D5. We tested the proportional-hazards assumption in these models by including an interaction term between lymphopenia trajectory group and log-transformed follow-up time.

The potential for ALC missingness to have biased results was examined by performing two sensitivity analyses, one assuming all participants with missing D5 ALC had lymphopenia, and the other assuming all participants with missing D5 ALC did not have lymphopenia and we repeated all outcome analyses to assess the robustness of our results.

### Statistical analysis: demographic, clinical and laboratory variables associated with lymphopenia

We evaluated the relationship between factors associated with lymphopenia at D0, new lymphopenia at D5 compared to no lymphopenia, and persistent lymphopenia at D5 compared to resolved lymphopenia using logistic regression. All covariates were D0 values unless otherwise noted. Models for D0 lymphopenia were adjusted for age, sex, race/ethnicity, residence, geographical region, infection period, baseline pulmonary status, N-Ag, SARS-CoV-2 viral load, eGFR, CRP, IL-6, and D-dimer, and use of corticosteroids, immunomodulators, and remdesivir. Models for new lymphopenia and resolved lymphopenia were additionally adjusted for randomized treatment group, and D5 use of corticosteroids, immunomodulators, and remdesivir. The adjusted relationships between COVID-related medications assessed 24 h prior to randomization, 24 h prior to D5, and at either time point and lymphopenia trajectory were displayed using forest plots.

Longitudinal plots of laboratory measurements were presented by lymphopenia trajectory group. Plasma N-Ag, CRP, IL-6, and D-dimer values were non-normally distributed and thus log-transformed for analyses, and back-transformed to the original scale for results reporting; thus, these biomarker levels were summarized by geometric means. Anti-N and anti-S Ab neutralizing activity were analyzed on the original scale and summarized as means. Longitudinal differences among lymphopenia trajectory groups were assessed using linear mixed effects models for each laboratory measurement with fixed effects for lymphopenia trajectory group, study day (categorical variable), baseline oxygen requirement, and the study day by lymphopenia trajectory group interaction, and random intercepts by participant. Global 3 degree of freedom tests were conducted to test for a difference among the 4 lymphopenia groups in biomarker levels across time points. Additionally, contrasts were tested for the pairwise comparisons of new lymphopenia vs. no lymphopenia and for persistent lymphopenia vs. resolved lymphopenia.

All statistical analyses were performed using SAS Version 9.4 (SAS institute, Cary NC). All tests were two-sided with *p* < 0.05 considered statistically significant. All analyses were performed for both lymphopenia and severe lymphopenia.

## Results

### TICO cohort baseline summary characteristics defined by lymphopenia trajectory

Among 2579 TICO participants with a D0 ALC value (Figure E1), 1426 (55.3%) had lymphopenia (Table E1) of whom 636 (44.6%) had severe lymphopenia (Table E2). 2105 of 2579 (81.6%) participants had a D5 ALC value (reasons for missingness Table E3), defining ALC trajectory as 38.9% (*n* = 819) with no lymphopenia, 32.6% (*n* = 686) with resolved lymphopenia, 4.7% (*n* = 101) with new lymphopenia, and 23.7% (*n* = 499) with persistent lymphopenia (Table 1). Compared to participants with no lymphopenia and resolved lymphopenia, participants with persistent or new lymphopenia were on average more commonly older, male, infected between July and December 2021 (delta variant predominant), had more vaccine doses (reflecting the more recent era in which they were enrolled), had a history of renal impairment, N Ag >/=1000 ng/L, higher oxygen requirement, eGFR < 60 ml/min/1.73m^2^, CRP > 7.5 mg/L, and IL-6 > 5.8 ng/L, and those with new lymphopenia had the highest proportion of participants with IL-6 > 5.8 ng/ml (Table 1). When trajectory groups were defined by severe lymphopenia, similar characteristic trends were observed (Table E4). Heterogeneity was seen among groups with respect to markers of end organ dysfunction, including D0 (Figure E2) and D5 (Figure E3) oxygen requirements, and D0 (Figure E4) and D5 (Figure E5) eGFR measurements. Although higher oxygen requirements and lower eGFR were proportionally higher in the groups with new or persistent lymphopenia/severe lymphopenia both at D0 and D5, all groups included participants with these findings.

**Table 1:** TICO cohort baseline summary characteristics by lymphopenia^a^ trajectory

	**No lymphopenia** **(*n*= 819)**		**Resolved lymphopenia** **(*n*= 686)**		**New lymphopenia** **(*n*=101)**		**Persistent lymphopenia** **(*n*= 499)**	
	**N**	**(%)**	**N**	**(%)**	**N**	**(%)**	**N**	**(%)**
**Age - med. (IQR) years**	54 (43 - 65)		55 (46 - 66)		61 (52 - 69)		64 (54 - 72)	
18-39 years	146	17.8	90	13.1	5	5.0	36	7.2
40-49 years	177	21.6	141	20.6	14	13.9	58	11.6
50-59 years	196	23.9	193	28.1	28	27.7	99	19.8
60-69 years	162	19.8	133	19.4	29	28.7	152	30.5
70-79 years	100	12.2	92	13.4	17	16.8	107	21.4
≥ 80 years	38	4.6	37	5.4	8	7.9	47	9.4
**Sex**								
Male	424	51.8	400	58.3	60	59.4	328	65.7
Female	395	48.2	286	41.7	41	40.6	171	34.3
**Race/ethnicity**								
Asian	33	4.0	39	5.7	9	8.9	25	5.0
Black	217	26.5	158	23.0	17	16.8	106	21.2
Hispanic	159	19.4	127	18.5	25	24.8	77	15.4
White	380	46.4	341	49.7	45	44.6	277	55.5
Other	30	3.7	21	3.1	5	5.0	14	2.8
**Region**								
United States	560	68.4	536	78.1	78	77.2	414	83.0
Europe	174	21.2	113	16.5	13	12.9	59	11.8
Africa	75	9.2	23	3.4	6	5.9	14	2.8
Asia	10	1.2	14	2.0	4	4.0	12	2.4
**Residence**								
Independent	779	95.1	643	93.7	98	97.0	466	93.4
Other	40	4.9	43	6.3	3	3.0	33	6.6
**Date of infection**								
Pre 2021	110	13.4	114	16.6	14	13.9	83	16.6
Jan-Jun 2021	334	40.8	291	42.4	30	29.7	181	36.3
Jul-Dec 2021	375	45.8	281	41.0	57	56.4	235	47.1
**Symptom duration - med. (IQR) days**	8 (6 - 10)		8 (6 - 10)		8 (5 - 10)		8 (6 - 9)	
< 5	120	14.7	99	14.4	22	21.8	73	14.6
5 - 7	233	28.4	192	28.0	20	19.8	156	31.3
8 - 10	352	43.0	286	41.7	40	39.6	201	40.3
> 10	114	13.9	109	15.9	19	18.8	69	13.8
**# vaccine doses**								
0	680	83.7	589	86.1	82	82.8	376	76.4
1	54	6.7	44	6.4	4	4.0	43	8.7
2	78	9.6	51	7.5	13	13.1	73	14.8
**Quanterix Ag - med. (IQR) ng/L**	785 (79 - 3073)		1595 (360 - 4655)		1733 (543 - 6095)		3486 (884 - 8611)	
1000+	358	44.7	412	60.8	69	70.4	352	73.3
< 1000	443	55.3	266	39.2	29	29.6	128	26.7
**SARS-CoV-2 viral load**								
Negative	126	15.9	89	13.3	7	7.2	42	8.6
< 35,000 copies/mL	358	45.3	310	46.5	47	48.5	170	35.0
35,000+ copies/mL	306	38.7	268	40.2	43	44.3	274	56.4
**Anti-spike Ab**								
Positive	440	54.9	356	52.5	50	51.0	202	42.1
Negative	361	45.1	322	47.5	48	49.0	278	57.9
**Anti-N Ab**								
Positive	520	64.8	435	64.2	57	58.2	271	56.5
Negative	282	35.2	243	35.8	41	41.8	209	43.5
**Asthma**								
Yes	86	10.5	58	8.5	6	5.9	46	9.2
No	733	89.5	628	91.5	95	94.1	453	90.8
**COPD**								
Yes	51	6.2	36	5.2	5	5.0	34	6.8
No	768	93.8	650	94.8	96	95.0	465	93.2
**Diabetes**								
Yes	195	23.8	188	27.4	37	36.6	161	32.3
No	624	76.2	498	72.6	64	63.4	338	67.7
**Heart failure**								
Yes	21	2.6	29	4.2	6	5.9	33	6.6
No	798	97.4	657	95.8	95	94.1	466	93.4
**Hypertension**								
Yes	318	38.8	311	45.3	50	49.5	271	54.3
No	501	61.2	375	54.7	51	50.5	228	45.7
**Renal impairment**								
Yes	43	5.3	61	8.9	14	13.9	94	18.8
No	776	94.7	625	91.1	87	86.1	405	81.2
**BMI - med. (IQR)**	31 (27 - 36)		31 (27 - 36)		30 (26 - 35)		29 (25 - 34)	
< 18.5 (underweight)	23	2.8	12	1.8	1	1.0	6	1.2
18.5-24.9 (healthy)	117	14.3	99	14.5	16	15.8	114	22.9
25-29.9 (overweight)	221	27.1	201	29.4	33	32.7	156	31.3
30-39.9 (obese)	324	39.7	280	41.0	39	38.6	169	33.9
≥ 40 (morbidly obese)	131	16.1	91	13.3	12	11.9	53	10.6
**Immunomodulators**								
Yes	46	5.6	49	7.1	7	6.9	48	9.6
No	773	94.4	637	92.9	94	93.1	451	90.4
**Corticosteroids**								
Yes	511	62.4	500	72.9	70	69.3	369	73.9
No	308	37.6	186	27.1	31	30.7	130	26.1
**Remdesivir prior to rand.**								
Yes	472	57.6	421	61.4	63	62.4	327	65.5
No	347	42.4	265	38.6	38	37.6	172	34.5
**Pulmonary status**								
No O2	233	28.4	150	21.9	19	18.8	96	19.2
O2 < 4 L/min	308	37.6	265	38.6	24	23.8	141	28.3
O2 ≥ 4 L/min	224	27.4	199	29.0	29	28.7	173	34.7
Non-invasive vent./HFNC	54	6.6	72	10.5	29	28.7	89	17.8
**Borg Dyspnea Scale**								
0-2 (nothing to slight)	374	49.4	290	45.5	42	46.2	212	45.0
3-4 (mod-somewhat severe)	249	32.9	206	32.3	31	34.1	148	31.4
5-10 (severe-maximal)	134	17.7	142	22.3	18	19.8	111	23.6
**NEWS**								
< 2	111	13.6	73	10.7	14	13.9	39	7.8
2-3	281	34.4	209	30.7	24	23.8	148	29.8
4-5	259	31.7	232	34.1	32	31.7	161	32.4
≥ 6	165	20.2	167	24.5	31	30.7	149	30.0
**Platelets – med. (IQR) x10**^**9**^**/L**	225 (178 - 393)		205 (166 - 262)		203 (159 - 260)		185 (134 - 237)	
**Hemoglobin – med. (IQR) g/dL**	13.5 (12.3 - 14.5)		13.2 (12.1 - 14.5)		13.3 (11.9 - 14.3)		12.7 (11.1 - 14.0)	
**Serum creatinine** ^b^ ** - med. (IQR) ** **mg/dL**	0.80 (0.66 - 1.00)		0.84 (0.70 - 1.05)		0.88 (0.70 - 1.20)		0.97 (0.74 - 1.40)	
< 1.1	656	80.2	534	78.0	70	69.3	298	59.7
1.1-1.5	103	12.6	101	14.7	15	14.9	101	20.2
> 1.5	59	7.2	50	7.3	16	15.8	100	20.0
**eGFR** ^b^ ** - med. (IQR)**	96 (76 - 111)		94 (74 - 108)		88 (61 - 102)		80 (50 - 98)	
< 60	103	12.6	105	15.3	25	24.8	162	32.5
≥ 60	715	87.4	580	84.7	76	75.2	337	67.5
**CRP** ^b^ ** - med. (IQR) mg/dL**	2.4 (1.2 - 4.7)		3.4 (1.7 - 5.7)		3.3 (1.7 - 5.6)		4.1 (2.0 - 6.6)	
< 5	586	77.3	432	68.8	64	73.6	265	60.0
5-7.5	90	11.9	101	16.1	7	8.0	90	20.4
> 7.5	82	10.8	95	15.1	16	18.4	87	19.7
**IL-6** ^c^ ** - med (IQR) ng/L**	5.2 (2.1 - 13.6)		4.9 (2.1 - 12.0)		9.2 (5.8 - 24.6)		9.7 (3.9 - 20.5)	
≤ 5.8	417	53.1	364	55.1	24	25.8	159	34.1
> 5.8	369	46.9	297	44.9	69	74.2	307	65.9
**D-dimer** ^c^ ** – med (IQR) mg/L**	0.85 (0.57 - 1.37)		0.94 (0.64 - 1.45)		1.07 (0.68 - 1.63)		1.09 (0.76 - 1.84)	
≤ 0.93	445	56.6	329	49.8	39	41.9	175	37.6
> 0.93	341	43.4	332	50.2	54	58.1	291	62.4

*IQR *interquartile range, *Ag* antigen, *Ab* antibody, *COPD* chronic obstructive pulmonary disease, *BMI* body mass index, *HFNC* high flow nasal canula, *NEWS* National Early Warning Score, *eGFR* estimated glomerular filtration rate, *CRP* C-reactive protein, *IL-6* interleukin 6

Percentages are calculated among those with available data for each variable

^a^Defined as lymphocyte count < 0.9

^b^Categories defined by clinically relevant cutoffs

^c^Categories defined by median of population at Day 0

### Relationship of lymphopenia with participant outcomes

D0 lymphopenia and severe lymphopenia were associated with increased mortality (aHR 1.48; 1.08, 2.05; aHR 1.60; 1.20, 2.14) and reduced sustained recovery (aRRR 0.90; 0.82, 0.99; aRRR 0.78; 0.70, 0.87) (Table 2). Neither D0 lymphopenia nor D0 severe lymphopenia were associated with increased risk of secondary infections (Table 3).

**Table 2:** Lymphopenia association with outcomes

**Mortality**				
**Day 0 lymphopenia (ALC<0.9)**	**N Pts.**	**N (%) Deaths**	**aHR**^**a**^ **(95% CI)**	***p*** **-value**
< 0.9	1426	191 (13.4)	1.48 [1.08, 2.05]	0.016
≥ 0.9	1153	66 (5.7)	(ref.)	
**Day 0 severe lymphopenia (ALC<0.56)**	**N Pts.**	**N (%) Deaths**	**aHR**^**a**^ **(95% CI)**	***p*** **-value**
< 0.56	636	115 (18.1)	1.60 [1.20, 2.14]	0.001
≥ 0.56	1943	142 (7.3)	(ref.)	
**Lymphopenia trajectory groups**	**N Pts.**	**N (%) Deaths**	**aHR**^**a**^ **(95% CI)**	***p*** **-value**
No lymphopenia	819	35 (4.3)	(ref.)	
Resolved lymphopenia	684	39 (5.7)	1.18 [0.72, 1.95]	0.52
New lymphopenia	101	13 (12.9)	1.91 [0.96, 3.81]	0.07
Persistent lymphopenia	497	120 (24.1)	2.68 [1.71, 4.19]	<0.001
**Severe lymphopenia trajectory groups**	**N Pts.**	**N (%) Deaths**	**aHR**^**a**^ **(95% CI)**	***p*** **-value**
No severe lymphopenia	1484	89 (6.0)	(ref.)	
Resolved severe lymphopenia	350	35 (10.0)	1.18 [0.76, 1.84]	0.46
New severe lymphopenia	93	23 (24.7)	1.79 [1.03, 3.13]	0.040
Persistent severe lymphopenia	174	60 (34.5)	3.05 [2.05, 4.53]	<0.001
**Recovery**				
**Day 0 lymphopenia (ALC<0.9)**	**N Pts.**	**N (%) Recovered**	**aRRR**^**b**^ **(95% CI)**	***p*** **-value**
< 0.9	1426	1170 (82.0)	0.90 [0.82, 0.99]	0.033
≥ 0.9	1153	1036 (89.9)	(ref.)	
**Day 0 severe lymphopenia (ALC<0.56)**	**N Pts.**	**N (%) Recovered**	**aRRR**^**b**^ **(95% CI)**	***p*** **-value**
< 0.56	636	483 (75.9)	0.78 [0.70, 0.87]	<0.001
≥ 0.56	1943	1723 (88.7)	(ref.)	
**Lymphopenia trajectory groups**	**N Pts.**	**N (%) Recovered**	**aRRR**^**b**^ **(95% CI)**	***p*** **-value**
No lymphopenia	819	760 (92.8)	(ref.)	
Resolved lymphopenia	684	631 (92.3)	1.05 [0.94, 1.18]	0.39
New lymphopenia	101	82 (81.2)	0.77 [0.59, 0.99]	0.043
Persistent lymphopenia	497	344 (69.2)	0.61 [0.53, 0.71]	<0.001
**Severe lymphopenia trajectory groups**	**N Pts.**	**N (%) Recovered**	**aRRR**^**b**^ **(95% CI)**	***p*** **-value**
No severe lymphopenia	1484	1351 (91.0)	(ref.)	
Resolved severe lymphopenia	350	305 (87.1)	0.87 [0.76, 0.99]	0.037
New severe lymphopenia	93	66 (71.0)	0.67 [0.51, 0.88]	0.004
Persistent severe lymphopenia	174	95 (54.6)	0.47 [0.37, 0.59]	<0.001

Day 0 lymphopenia and Day 0 severe lymphopenia models include 2236 participants with all covariates available. Lymphopenia and severe lymphopenia models include 1861 participants with Day 5 ALC and all covariates available

^a^Hazard ratio for death adjusted for age, gender, race/ethnicity, residence, geographical region, date of infection, baseline pulmonary status, Quanterix Ag, SARS-CoV-2 viral load, eGFR, CRP, IL-6, and D-dimer

^b^Recovery rate ratio for sustained recovery adjusted for age, gender, race/ethnicity, residence, geographical region, date of infection, baseline pulmonary status, Quanterix Ag, SARS-CoV-2 viral load, eGFR, CRP, IL-6, and D-dimer

**Table 3:** Lymphopenia and severe lymphopenia association with secondary infections

	**N Pts.**	**N (%) with Infection**	**aHR**^**a**^ **(95% CI)**	***p*** **-value**
**ALC < 0.9**				
**Day 0 lymphopenia**				
< 0.9	1426	144 (10.1)	1.23 [0.88, 1.71]	0.22
0.9+	1153	63 (5.5)	(ref.)	
**Lymphopenia trajectory groups**				
No lymphopenia	808	30 (3.7)	(ref.)	
Resolved lymphopenia	678	23 (3.4)	0.75 [0.42, 1.33]	0.32
New lymphopenia	96	11 (11.5)	1.99 [0.96, 4.14]	0.06
Persistent lymphopenia	470	78 (16.6)	2.43 [1.48, 3.98]	<0.001
**ALC < 0.56**				
**Day 0 severe lymphopenia**				
< 0.56	636	84 (13.2)	1.36 [0.99, 1.85]	0.054
0.56+	1943	123 (6.3)	(ref.)	
**Severe lymphopenia trajectory groups**				
No severe lymphopenia	1460	63 (4.3)	(ref.)	
Resolved severe lymphopenia	345	26 (7.5)	1.08 [0.65, 1.78]	0.77
New severe lymphopenia	85	18 (21.2)	2.29 [1.24, 4.21]	0.008
Persistent severe lymphopenia	162	35 (21.6)	3.21 [2.01, 5.14]	<0.001

Day 0 lymphopenia and Day 0 severe lymphopenia models include 2236 participants with all covariates available. Lymphopenia and severe lymphopenia models include 1816 participants who did not have a secondary infection in the first 5 days and have Day 5 ALC and all covariates available

^a^Hazard ratio for secondary infection adjusted for age, gender, race/ethnicity, residence, geographical region, date of infection, baseline pulmonary status, Quanterix Ag, SARS-CoV-2 viral load, eGFR, CRP, IL-6, and D-dimer

**Fig. 1:**
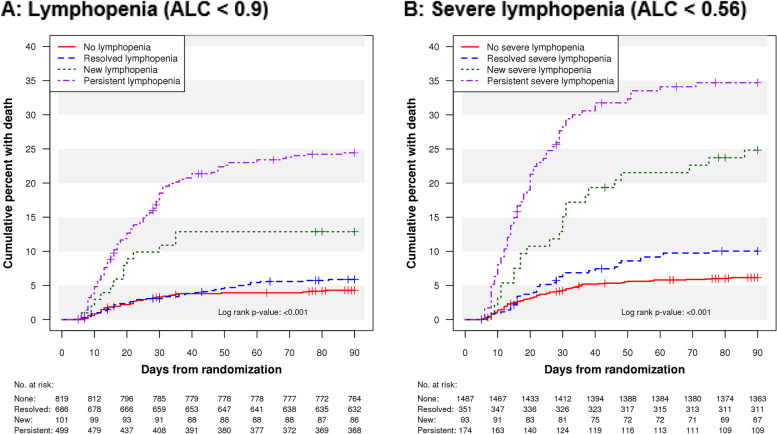
Time to death Kaplan-Meier curves by lymphopenia trajectory groups (**A**) and severe lymphopenia trajectory groups (**B**)

In terms of lymphopenia trajectory, participants with no lymphopenia and resolved lymphopenia had the lowest 90-day mortality, while mortality was higher among participants with new lymphopenia and persistent lymphopenia (log rank composite *p* < 0.001) (Fig. 1A). Mortality curve differences between groups appeared by day 10, and the groups with persistent or new lymphopenia had the worst prognosis with nearly 25% and 13% mortality by day 90, respectively, markedly higher than the no and resolved lymphopenia groups (mortality ~ 5%). Similar patterns emerged for sustained recovery, and participants with new or persistent lymphopenia exhibited the least sustained recovery (Figure E6). More pronounced, but similar, trends were seen in mortality and sustained recovery among groups using the severe lymphopenia cutoff (Fig. 1B, Figure E7).

In adjusted analyses of lymphocyte trajectory groups, persistent lymphopenia was associated with increased mortality (aHR 2.68; 1.71, 4.19) while resolved lymphopenia and new lymphopenia were not (aHR 1.18; 0.72, 1.95; aHR 1.91; 0.96, 3.81), when compared to participants with no lymphopenia (Table 2). Sustained recovery was less common among participants with persistent lymphopenia or new lymphopenia (aRR 0.61; 0.53, 0.71; aRRR 0.77; 0.59, 0.99), but not with resolved lymphopenia (aRRR 1.05; 0.94, 1.18), when compared to no lymphopenia. The persistent lymphopenia group was more likely to experience a secondary infection when compared to no lymphopenia (aHR 2.43; 1.48, 3.98) (Table 3). The HRs for mortality and secondary infections and the RRRs for recovery did not vary significantly over the follow-up period (*p* = 0.20, 0.75, and 0.41, respectively) for proportional hazards.

When categorizing participants by severe lymphopenia, mortality and recovery patterns were similar, though participants with new severe lymphopenia also had increased mortality risk (aHR 1.79, 1.03, 3.13) compared to participants with no severe lymphopenia (Table 2). Participants with new severe lymphopenia (aHR 2.29; 1.24, 4.21), and persistent severe lymphopenia (aHR 3.21; 2.01, 5.14) were at increased risk for secondary infections when compared to no severe lymphopenia (Table 3).

Results were generally unchanged when assuming all 474 participants with missing D5 ALC had lymphopenia or severe lymphopenia (Table E5) and when assuming they did not have lymphopenia or severe lymphopenia (Table E6).

### Demographic, clinical and laboratory variables associated with lymphopenia

D0 lymphopenia (Table 4, Supplemental Tables E7-11) was associated with older age, male sex, white race, normal BMI, location in the US, history of renal impairment, history of malignancy, use of aspirin, and use of antirejection medications. When looking at COVID-19 specific factors, infection prior to 2021 compared to July-December 2021, and symptom duration 8–10 days compared to less than 5 days, NIV/HFNC, eGFR < 60, higher N-Ag quanterix, higher CRP, and D-dimer > 0.93 were associated with D0 lymphopenia. Similar findings were seen for severe lymphopenia (Table E12) with some differences. Notably, IL-6 ≤ 5.8 was associated with D0 lymphopenia but not severe lymphopenia. Among COVID-specific treatments assessed at D0, corticosteroids were associated with increased odds of D0 lymphopenia and severe lymphopenia (Table 4, Table E13, and Table E14).

**Table 4:** Factors associated with Day 0 lymphopenia, new (Day 5) lymphopenia, or resolved (Day 5) lymphopenia

	**Day 0 lymphopenia**		**New lymphopenia compared to no lymphopenia**		**Resolved lymphopenia compared to persistent lymphopenia**	
	**N Pts.**	**aOR (95% CI)**^**a**^	**N Pts.**	**aOR (95% CI)**^**b**^	**N Pts.**	**aOR (95% CI)**^**b**^
**Age**						
18-39 years	352	(ref.)	151	(ref.)	126	(ref.)
40-49 years	476	1.18 (0.86 - 1.61)	191	2.90 (0.91 - 9.26)	199	0.91 (0.52 - 1.59)
50-59 years	618	1.30 (0.96 - 1.76)	224	4.77 (1.57 - 14.50)**	292	0.86 (0.51 - 1.46)
60-69 years	559	1.39 (1.01 - 1.92)*	191	3.84 (1.23 - 11.96)*	285	0.43 (0.26 - 0.73)*
70-79 years	399	1.38 (0.97 - 1.96)	117	5.33 (1.60 - 17.72)**	199	0.52 (0.29 - 0.91)*
≥ 80 years	175	1.64 (1.02 - 2.61)*	46	3.46 (0.71 - 16.93)	84	0.56 (0.28 - 1.15)
**Race/Ethnicity**						
White	1280	(ref.)	425	(ref.)	618	(ref.)
Asian	119	1.20 (0.71 - 2.03)	42	2.56 (0.74 - 8.90)	64	1.13 (0.51 - 2.50)
Black	619	0.84 (0.65 - 1.09)	234	0.44 (0.19 - 1.06)	264	1.26 (0.86 - 1.83)
Hispanic	474	0.70 (0.54 - 0.90)**	184	1.33 (0.65 - 2.70)	204	1.18 (0.79 - 1.79)
Other	87	0.65 (0.40 - 1.06)	35	0.92 (0.25 - 3.44)	35	1.29 (0.57 - 2.93)
**Sex**						
Male	1484	(ref.)	484	(ref.)	728	(ref.)
Female	1095	0.67 (0.56 - 0.80)***	436	0.71 (0.42 - 1.19)	457	1.42 (1.07 - 1.89)*
**Region**						
United States	2019	(ref.)	638	(ref.)	950	(ref.)
Europe	389	0.68 (0.52 - 0.88)**	187	0.44 (0.19 - 1.00)	172	1.23 (0.81 - 1.86)
Africa	129	0.39 (0.24 - 0.62)***	81	1.62 (0.43 - 6.07)	37	0.95 (0.40 - 2.22)
Asia	42	0.76 (0.33 - 1.77)	14	1.13 (0.18 - 7.19)	26	0.90 (0.28 - 2.87)
**Date of Infection**						
Pre 2021	401	1.49 (1.09 - 2.04)*	124	0.61 (0.23 - 1.60)	197	0.82 (0.52 - 1.30)
Jan-Jun 2021	1034	1.08 (0.88 - 1.33)	364	0.87 (0.48 - 1.59)	472	1.16 (0.83 - 1.62)
Jul-Dec 2021	1144	(ref.)	432	(ref.)	516	(ref.)
**Symptom duration (days)**						
< 5	393	(ref.)	142	(ref.)	172	(ref.)
5 - 7	749	1.11 (0.83 - 1.48)	253	0.41 (0.19 - 0.90)^*^	348	1.01 (0.65 - 1.59)
8 - 10	1070	1.33 (1.01 - 1.76)*	392	0.45 (0.22 - 0.94)*	487	0.91 (0.58 - 1.41)
> 10	367	1.39 (0.99 - 1.97)	133	0.93 (0.38 - 2.30)	178	1.01 (0.59 - 1.73)
**Quanterix Ag (ng/L)**						
< 200	598	(ref.)	272	(ref.)	198	(ref.)
200 - 1499	670	1.52 (1.18 - 1.95)**	269	1.42 (0.62 - 3.21)	284	0.94 (0.59 - 1.49)
1500 - 4499	583	2.27 (1.73 - 2.98)***	178	2.52 (1.10 - 5.74)*	290	0.83 (0.53 - 1.32)
≥ 4500	651	2.66 (1.99 - 3.55)***	180	2.03 (0.87 - 4.75)	386	0.62 (0.39 - 0.99)*
1000+	1434	2.02 (1.65 - 2.46)***	427	2.02 (1.12 - 3.64)*	764	0.83 (0.61 - 1.14)
< 1000	1068	(ref.)	472	(ref.)	394	(ref.)
**BMI**						
< 18.5 (underweight)	47	0.70 (0.35 - 1.40)	24	0.44 (0.05 - 3.92)	18	2.77 (0.87 - 8.84)
18.5-24.9 (healthy)	422	(ref.)	133	(ref.)	213	(ref.)
25-29.9 (overweight)	743	0.71 (0.53 - 0.94)*	254	1.08 (0.47 - 2.53)	357	1.59 (1.05 - 2.39)*
30-39.9 (obese)	985	0.57 (0.43 - 0.75)***	363	1.08 (0.48 - 2.47)	449	2.09 (1.38 - 3.16)***
≥ 40 (morbidly obese)	374	0.54 (0.38 - 0.77)***	143	1.01 (0.35 - 2.93)	144	1.90 (1.11 - 3.25)*
**Heart failure**						
Yes	114	1.29 (0.81 - 2.08)	27	5.16 (1.58 - 16.77)**	62	1.09 (0.60 - 1.97)
No	2465	(ref.)	893	(ref.)	1123	(ref.)
**Renal impairment**						
Yes	257	1.75 (1.20 - 2.56)**	57	1.49 (0.58 - 3.86)	155	0.69 (0.43 - 1.11)
No	2322	(ref.)	863	(ref.)	1030	(ref.)
**Non-HIV immune suppression**						
Yes	80	1.57 (0.91 - 2.71)	20	0.51 (0.08 - 3.12)	50	0.49 (0.25 - 0.98)*
No	2499	(ref.)	900	(ref.)	1135	(ref.)
**Malignancy**						
Yes	105	2.05 (1.23 - 3.39)**	23	0.19 (0.02 - 1.71)	64	0.31 (0.16 - 0.59)***
No	2474	(ref.)	897	(ref.)	1121	(ref.)
**Pulmonary status**						
No O2	644	(ref.)	252	(ref.)	246	(ref.)
O2 < 4 L/min	932	1.04 (0.81 - 1.34)	332	0.84 (0.37 - 1.92)	406	1.31 (0.87 - 1.97)
O2 ≥ 4 L/min	719	1.28 (0.97 - 1.68)	253	1.38 (0.60 - 3.17)	372	0.93 (0.61 - 1.43)
NIV/HFNC	284	1.47 (1.00 - 2.17)*	83	5.04 (1.90 - 13.35)**	161	0.71 (0.40 - 1.26)
**Serum creatinine ** **mg/dL**						
< 1.1	1902	(ref.)	726	(ref.)	832	(ref.)
1.1-1.5	400	1.22 (0.94 - 1.59)	118	1.30 (0.57 - 2.92)	202	0.67 (0.46 - 0.98)*
> 1.5	274	1.32 (0.95 - 1.84)	75	4.07 (1.77 - 9.32)***	150	0.30 (0.19 - 0.49)***
**eGFR**						
< 60	497	1.31 (1.01 - 1.69)*	128	2.72 (1.36 - 5.42)**	267	0.48 (0.34 - 0.69)***
≥ 60	2079	(ref.)	791	(ref.)	917	(ref.)
**CRP (mg/L)**						
< 5	1633	(ref.)	650	(ref.)	697	(ref.)
5-7.5	351	1.48 (1.14 - 1.93)**	97	0.49 (0.19 - 1.24)	191	0.88 (0.61 - 1.26)
> 7.5	330	1.32 (1.01 - 1.73)*	98	1.01 (0.51 - 2.02)	182	1.02 (0.69 - 1.49)
**IL-6 (ng/L)**						
≤ 5.8	1207	(ref.)	441	(ref.)	523	(ref.)
> 5.8	1227	0.66 (0.54 - 0.81)***	438	2.33 (1.28 - 4.23)**	604	0.67 (0.49 - 0.91)**
**D-dimer (mg/L)**						
≤ 0.93	1213	(ref.)	484	(ref.)	504	(ref.)
> 0.93	1221	1.42 (1.18 - 1.72)***	395	1.11 (0.64 - 1.95)	623	0.81 (0.61 - 1.08)
**Corticosteroid use**						
Yes	1756	1.31 (1.05 - 1.62)*	581	1.27 (0.65 - 2.49)	869	1.33 (0.93 - 1.89)
No	823	(ref.)	339	(ref.)	316	(ref.)
**Aspirin use**						
Yes	385	1.38 (1.06 - 1.81)*	101	2.57 (1.26 - 5.26)**	210	1.05 (0.73 - 1.51)
No	2194	(ref.)	819	(ref.)	975	(ref.)
**Antirejection medication**						
Yes	101	2.69 (1.53 - 4.73)***	17	5.50 (1.54 - 19.60)**	66	0.27 (0.14 - 0.54)***
No	2478	(ref.)	903	(ref.)	1119	(ref.)

*Ag* antigen, *BMI* body mass index, *eGFR* estimated glomerular filtration rate, *CRP* C-reactive protein, *IL-6* interleukin 6

Day 0 models include 2236 participants with all covariates available. New lymphopenia compared to no lymphopenia models include 817 participants and resolved lymphopenia compared to persistent lymphopenia models include 1039 participants

*Significant *p*-values are denoted as follows next to parentheses: * *p*-value < 0.05, ** *p*-value < 0.01, *** *p*-value < 0.001

^a^Adjusted for age, sex, race/ethnicity, residence, geographical region, date of infection, baseline pulmonary status, Quanterix Ag, SARS-CoV-2 viral load, eGFR, CRP, IL-6, D-dimer, corticosteroid use, immunomodulator use, and remdesivir use

^b^Adjusted for age, sex, race/ethnicity, residence, geographical region, date of infection, baseline pulmonary status, Quanterix Ag, SARS-CoV-2 viral load, eGFR, CRP, IL-6, D-dimer, D0 and D5 corticosteroid use, D0 and D5 immunomodulator use, D0 and D5 remdesivir use, and randomized treatment group

When considering lymphopenia trajectory, compared to those with no lymphopenia, new lymphopenia was associated with older age, history of heart failure, location in the US, use of aspirin, and use of antirejection medications (Table 4). Compared to resolved lymphopenia persistent lymphopenia was associated with older age, male sex, normal BMI, history of malignancy, history of non-HIV immune suppression, and use of anti-rejection medications. COVID-19 specific factors, including baseline markers of organ dysfunction, host immune response and viral replication, also differed between lymphopenia trajectory groups. When compared to those with no lymphopenia, symptom duration < 5 days, NIV/HFNC, eGFR < 60, higher N-Ag quanterix, and IL-6 > 5.8 were associated with new lymphopenia. Compared to those with resolved lymphopenia, participants with persistent lymphopenia had increased odds of baseline IL-6 > 5.8, higher N-Ag quanterix, and an eGFR < 60. Similar findings were seen for severe lymphopenia (Table E12).

When assessing COVID-specific treatments at D0, therapeutic heparin was associated with resolved severe lymphopenia compared to persistent severe lymphopenia (Figure E9 and Table E14) and when assessing D5 medications, therapeutic heparin was associated with decreased odds of new lymphopenia compared to no lymphopenia (Figure E8 and Table E13). D5 corticosteroid use was associated with increased odds of persistent lymphopenia compared to resolved lymphopenia (Figure E8 and Table E13) and D5 immunomodulator use was associated with new severe lymphopenia when compared to no severe lymphopenia (Figure E9 and Table E14). Remdesivir and TICO investigational agents were not associated with lymphopenia trajectories (Figure E8 and E9).

**Fig. 2:**
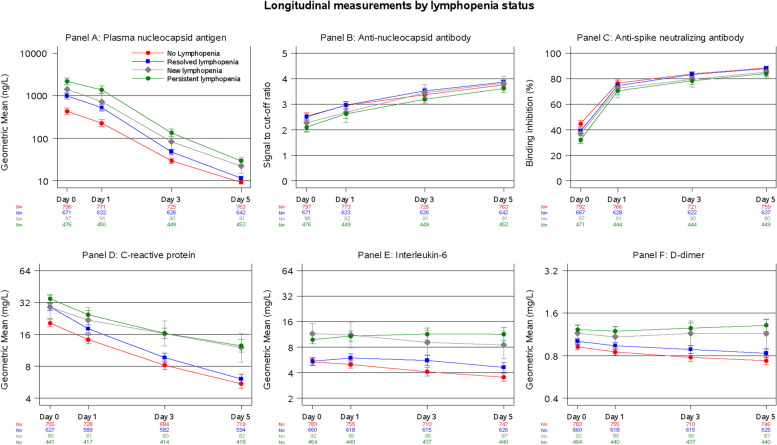
Biomarker measurements at Days 0, 1, 3, and 5 by lymphopenia trajectory group. Plasma nucleocapsid antigen (Panel **A**), C-reactive protein (Panel **D**), interleukin-6 (Panel **E**), and D-dimer (Panel **F**) are non-normally distributed and are summarized by geometric means. Anti-nucleocapsid antibody (Panel **B**) and anti-spike neutralizing antibody (Panel **C**) are displayed as means on the original scale

When assessing serial measurements of other biomarkers and comparing amongst all four lymphopenia trajectory groups, there were qualitative differences in biomarker trajectory, especially for plasma N-Ag quanterix, CRP, IL-6, and D-dimer with higher values at day 3 or day 5 for new or persistent lymphopenia groups (Fig. 2). In addition to significant overall differences for each biomarker between all lymphocyte trajectory groups (*p* < 0.001), specific pairwise comparisons between new lymphopenia vs. no lymphopenia or persistent lymphopenia vs. resolved lymphopenia were also significantly different for each biomarker (*p* < 0.05). Similar findings were observed for severe lymphopenia trajectory groups (Figure E10), with visually wider curve separation at earlier timepoints and for specific biomarkers.

## Discussion

In this post hoc analysis of five TICO studies, we found that participants with lymphopenia or severe lymphopenia that was persistent or new at D5 had increased mortality, decreased recovery, and increased secondary infections when compared to those with no lymphopenia, while those with resolved lymphopenia did not. These results support accumulating data that ALC, a routinely measured laboratory variable, is correlated with severe COVID-19 outcomes. While most prior studies have focused on a single ALC measurement, our data is unique in that it suggests that a repeat ALC at D5 may be more predictive than a single value.

While lymphopenia has been described as a predictive biomarker in hospitalized COVID-19 patients, the causes are not well understood. One potential explanation is that SARS-CoV-2 infects and leads to loss of circulating lymphocytes through apoptosis, although data from in vitro studies are conflicting [21]. Another potential explanation is cytokine mediated cell death as high levels of pro-inflammatory cytokines have been linked to low ALC in COVID-19 patients [16, 22, 23]. IL-6, in particular, has been shown in vitro to induce T cell death [23] and interestingly was elevated in our cohort among those with persistent and new lymphopenia. Other potential mechanisms, including a dysregulated inflammatory response that impedes T cell proliferation or activation and contributes to persistence of infected T cells [24, 25], reduced thymic lymphocyte production [26], shortened telomeres impacting maintenance of the lymphocyte pool [27], and increased tissue extravasation or syncytia formation [28] in sites of ongoing infection may all contribute to lymphopenia. Additionally, in our study, antibodies were present even in those with lymphopenia, suggesting that B cells may be functional, although we were not able to evaluate this. In short, the mechanism of lymphopenia in hospitalized SARS-CoV-2 infection remains uncertain and broadening our understanding of the pathobiology that drives lymphopenia is crucial to provide input into these hypotheses coupled with in-depth laboratory measurements on specific lymphocyte profiles.

To our knowledge this study is the largest analysis of demographic, clinical, and laboratory characteristics associated with lymphopenia and lymphopenia trajectory in COVID-19. Most of the demographic and pre-existing clinical conditions described here have been linked to differences in lymphocyte count in the general population. For example, older age [29] and male sex [30] have known associations with lower ALC in healthy adults, while individuals who are immunosuppressed, have a malignancy, or are taking anti-rejection medications may have lower lymphocyte counts due to their underlying disease or disease intervention. All of these groups have worse COVID-19 outcomes [31–34], thus it may have been expected that these factors would be associated with new or persistent lymphopenia. We also identified new factors associated with new or persistent lymphopenia such as aspirin use, heart failure, and normal BMI that warrant further exploration. As lymphopenia in the general population has been associated with increased mortality independent of other risk factors [35] it is conceivable that ALC can be measured in target groups as a predictor for infection related outcomes [36] prior to the onset of illness. Studies aimed at better understanding immune function among patients predisposed to lymphopenia could provide insight into disease pathogenesis and give opportunity to bolster immunity ahead of COVID-19 infection.

The biological mechanism by which lymphopenia is associated with severe COVID-19 outcomes has not been established, however here we observed associations of new or persistent lymphopenia with clinical measurements of end-organ dysfunction and biomarkers suggestive of ineffective immunologic response. Association of higher oxygen requirements and lower eGFR with new or persistent lymphopenia suggest correlation to organ dysfunction, while higher IL-6 and higher viral load suggest an ineffective immunologic response. Serial trends in viral antigen, CRP and IL-6, provide support that new or persistent lymphopenia is associated with ongoing viral replication and inflammation. A recent paper by Michels et al. [37] divided lymphopenic hospitalized COVID-19 patients into three groups on the basis of coinciding clusters of other biomarkers. This allowed further phenotype assessment beyond lymphopenia, with one group deemed “inflammatory injurious” having a higher mortality rate than the other two. As this analysis relied on baseline values only, it would be interesting to assess such groups over time to see if serial ALC assessments can prognostically enrich for high-risk individuals independent of biomarkers not easily measured in clinical labs. Several other studies have suggested similarly that a depletion in lymphocytes is associated with delays in viral clearance and that skewed lymphocyte subset responses potentially cause inflammation without virologic control [10, 38]. More precise characterization of lymphocytes subsets and the quality of immunologic response beyond the limitations of our data is needed. Finally, participants with persistent lymphopenia or new severe lymphopenia had increased risk for secondary infections, suggesting that dysregulated immunity not only contributes to COVID-19 severity but also increases susceptibility to other infections. This observation warrants longer-term follow-up to assess the duration of lymphopenia and infection risk, as persistent immune dysfunction has been described after other infections, such as measles [39, 40]. Although many questions remain unanswered, trial design that incorporates biomarker-based assessments of immunologic phenotype warrants further discussion.

While we did not assess the benefit of COVID-19 therapeutics based on lymphopenia trajectory groups, we did note associations of heparin, corticosteroids and immunomodulators with lymphopenia trajectories. Analyses of other COVID-19 datasets have revealed differences in therapeutic response based on biomarkers. In a post hoc analysis of the Adaptive Covid-19 Treatment Trial, low ALC coupled with high absolute neutrophil count (ANC), low platelet count, and high oxygen requirements was able to predict differential response to an antiviral, remdesivir [41] and to an immunomodulator, baricitinib [10]. Similarly, limited retrospective data suggest that low ALC when coupled with other biomarkers can predict differential responses to corticosteroids [37]. There may also be opportunities to leverage trends to assess treatment response. For example, In the ACTT post hoc analysis of baricitinib response, differential increases in ALC and decreases in ANC were seen after 5 days of baricitinib treatment when compared to the control group [10]. A biomarker-based approach may better capture risk of severe outcomes, define immune phenotypes that benefit from therapeutics, and assess treatment responses in hospitalized COVID-19 patients.

This analysis has several limitations. First, this is a post hoc analysis assessing outcomes different from the primary TICO outcome. These outcomes were not adjusted for type one error and no adjustments were done for multiple comparisons. Second, D5 ALC was not available for 18% of the population. Although we attempted to account for this with a sensitivity analysis, this missingness may not have been at random and could have biased our results. Third, there were limits of data collection which included time points assessed for laboratory values and concomitant medications. Medications that may have been utilized for COVID-19 management were not specifically designated and thus alternative use indications might have been present. Laboratory values associated with COVID-19 outcomes in other studies, such as ANC, were not collected. In addition, the presence or absence of anti-S ab after D0 cannot be decoupled from TICO treatment in participants receiving them which likely impacted anti-S ab trajectories in biomarker analyses. Fourth, D0 values may have reflected variable timepoints in subject illness and, when assessing symptom duration, differences were noted for D0 and new lymphopenia but not persistent lymphopenia or with any severe lymphopenia group. Next, although the TICO dataset was largely representative of hospitalized COVID-19 patients from August 2020 through November 2021 these data may not be applicable to currently circulating variants or an immune experienced population. In addition, mechanically ventilated patients were excluded from the TICO studies and thus lymphopenia trajectory could not be assessed in this sickest group.

Despite these limitations, this post hoc analysis of the TICO dataset represents one of the largest efforts to evaluate the prognostic implications of lymphopenia trajectory in hospitalized COVID-19 patients. We show that those with persistent lymphopenia or new lymphopenia at D5 have worse outcomes than those with no lymphopenia or resolved lymphopenia. A better understanding of the underlying risk factors for and mechanisms that lead to the observed trajectories will help us to better understand COVID-19 disease pathogenesis and provide insight into better management strategies, especially for patients with comorbidities.

## Supplementary Information

Supplementary Material 1.

## Data Availability

Send requests for data or statistical code to the STRIVE Scientific Steering Committee at strive.science.program@vumc.org. These data will be made available to researchers whose proposed use of the data has been approved for a specified purpose by the Scientific Steering Committee.
