# Supplementary material for: Blood absolute lymphocyte count and trajectory are important in understanding severe COVID-19
Source: BMC Infect Dis. 2025 Jan 15;25:67. doi: 10.1186/s12879-024-10428-7 (PMC11734232; doi:10.1186/s12879-024-10428-7)
Supplement: Supplementary file 1 — Supplementary Material 1. [file 12879_2024_10428_MOESM1_ESM.docx]

**Online Data Supplement**

Blood absolute lymphocyte count and trajectory are important in understanding severe COVID-19

Authors:

Catharine I. Paules MD, Jacqueline A. Nordwall, Kathryn Shaw-Saliba, Judith A. Aberg, Edward M. Gardner, Anna L. Goodman, N. Kumarasamy, Shikha Vasudeva, David M. Vock, Crystal M. North, Jens Lundgren, Neil R. Aggarwal; for the STRIVE TICO Study Group

Contents

[Section 1: Supplemental Methods 4](#_Toc184297515)

[Eligibility Criteria 4](#_Toc184297516)

[Clinical Data 7](#_Toc184297517)

[Local Laboratory Measurements 8](#_Toc184297518)

[Central Laboratory Measurements 9](#_Toc184297519)

[Statistical Analysis 10](#_Toc184297520)

[Section 2: Supplemental Tables and Figures 12](#_Toc184297521)

[Figure E1. Flowchart of Participant Selection 12](#_Toc184297522)

[Table E1. Baseline characteristics by Day 0 Lymphopenia Status 14](#_Toc184297523)

[Table E2. Baseline characteristics by Day 0 Severe Lymphopenia Status 17](#_Toc184297524)

[Table E3. Reasons for Missing Day 5 ALC 20](#_Toc184297525)

[Table E4. Baseline characteristics by Severe Lymphopenia (< 0.56) Trajectory 21](#_Toc184297526)

[Figure E2 A/B: Oxygen requirements at D0 by Lymphopenia Trajectory Group and Severe Lymphopenia Trajectory Group 24](#_Toc184297527)

[Figure E3 A/B: Oxygen requirements at D5 by Lymphopenia Trajectory Group and Severe Lymphopenia Trajectory Group 25](#_Toc184297528)

[Figure E4 A/B: Estimated Glomerular Filtration Rate (eGFR) at D0 by Lymphopenia Trajectory Group and Severe Lymphopenia Trajectory Group 26](#_Toc184297529)

[Figure E5 A/B: Estimated Glomerular Filtration Rate (eGFR) at D5 by Lymphopenia Trajectory Group and Severe Lymphopenia Trajectory Group 27](#_Toc184297530)

[Table E5: Sensitivity analysis – Lymphopenia association with outcomes setting those with missing Day 5 Absolute Lymphocyte Count to having lymphopenia 28](#_Toc184297531)

[Table E6: Sensitivity analysis – Lymphopenia association with outcomes setting those with missing Day 5 Absolute Lymphocyte Count to not having lymphopenia 30](#_Toc184297532)

[Figure E6: Time to Recovery by lymphopenia trajectory groups (ALC < 0.9) 32](#_Toc184297533)

[Figure E7: Time to Recovery by severe lymphopenia trajectory groups (ALC < 0.56) 33](#_Toc184297534)

[Table E7: Demographic Factors associated with Day 0 Lymphopenia, New (Day 5) Lymphopenia, or Resolved (Day 5) Lymphopenia 34](#_Toc184297535)

[Table E8: COVID-19 Characteristics associated with Day 0 Lymphopenia, New (Day 5) Lymphopenia, or Resolved (Day 5) Lymphopenia 36](#_Toc184297536)

[Table E9: Comorbid Conditions associated with Day 0 Lymphopenia, New (Day 5) Lymphopenia, or Resolved (Day 5) Lymphopenia 38](#_Toc184297537)

[Table E10: Concomitant Medications associated with Day 0 Lymphopenia, New (Day 5) Lymphopenia, or Resolved (Day 5) Lymphopenia 40](#_Toc184297538)

[Table E11: COVID-19 Severity Factors associated with Day 0 Lymphopenia, New (Day 5) Lymphopenia, or Resolved (Day 5) Lymphopenia 42](#_Toc184297539)

[Table E12: Demographic, clinical, and COVID-19 related factors associated with Day 0 Severe Lymphopenia, New (Day 5) Severe Lymphopenia, or Resolved (Day 5) Severe Lymphopenia 44](#_Toc184297540)

[Table E13: COVID-19 Related Medications associated with Day 0 Lymphopenia, New (Day 5) Lymphopenia, or Resolved (Day 5) Lymphopenia 48](#_Toc184297541)

[Table E14: COVID-19 Related Medications associated with Day 0 Severe Lymphopenia, New (Day 5) Severe Lymphopenia, or Resolved (Day 5) Severe Lymphopenia 50](#_Toc184297542)

[Figure E8: Association of COVID-19 Related Medications with New lymphopenia when compared to no lymphopenia and resolved lymphopenia when compared to persistent lymphopenia 52](#_Toc184297543)

[Figure E9: Severe Lymphopenia association with COVID treatments 53](#_Toc184297544)

[Figure E10: Longitudinal biomarker measurements by severe lymphopenia trajectory groups 54](#_Toc184297545)

# Section 1: Supplemental Methods

## Eligibility Criteria

**Therapeutics for inpatients with COVID-19 (TICO) Master Protocol Platform Eligibility Criteria**

**Inclusion Criteria**

1. Age ≥ 18 years;
2. Informed consent by the patient or the patient’s legally authorized representative (LAR)
3. SARS-CoV-2 infection, documented by a nucleic acid test (NAT) or equivalent testing within 3 days prior to randomization OR documented by NAT or equivalent testing more than 3 days prior to randomization AND progressive disease suggestive of ongoing SARS-CoV-2 infection per the responsible investigator (For non-NAT tests, only those deemed with equivalent specificity to NAT by the protocol team will be allowed. A central list of allowed non-NAT tests will be maintained.)
4. Duration of symptoms attributable to COVID-19 ≤ 12 days per the responsible investigator.
5. Requiring admission for inpatient hospital acute medical care for clinical manifestations of COVID-19, per the responsible investigator, and NOT for purely public health or quarantine purposes.

**Exclusion Criteria**

1. Prior receipt of

- Any SARS-CoV-2 hIVIG, convalescent plasma from a person who recovered from COVID-19 or
- SARS-CoV-2 nMAb at any time prior to hospitalization

1. Not willing to abstain from participation in other COVID-19 treatment trials until after Day 5 (With the approval of study leadership, enrolment before or on Day 5 is permitted for individual trials.)
2. In the opinion of the responsible investigator, any condition for which, participation would not be in the best interest of the participant or that could limit protocol specified assessments.
3. Expected inability to participate in study procedures.

Prior to the initial futility assessment for an investigational agent, the following two additional exclusions (5 and 6) which define disease severity stratum 2 apply:

1. Presence at enrolment of any of the following:
   1. stroke
   2. meningitis
   3. encephalitis
   4. myelitis
   5. myocardial infarction
   6. myocarditis
   7. pericarditis
   8. symptomatic CHF (NYHA class III-IV)
   9. arterial or deep venous thrombosis or pulmonary embolism
2. Current requirement for any of the following:
   1. invasive mechanical ventilation
   2. ECMO
   3. mechanical circulatory support
   4. vasopressor therapy
   5. commencement of renal replacement therapy at this admission (i.e. not patients on chronic renal replacement therapy).

**Bamlanivimab-specific eligibility criteria**

1. Non-pregnant female participants who are of reproductive potential and male participants who are able to father a child must abstain from male/female sexual intercourse or agree to use two forms of effective contraception, where at least one form is highly effective (less than 1% failure rate), for the entirety of the study and for 90 days after investigational agent is administered.

Highly effective methods of contraception (less than 1% failure rate) include, but are not limited to:

- combination oral contraceptives
- implanted contraceptives
- intrauterine devices

Effective methods of contraception include, but are not limited to:

- diaphragms and cervical caps with spermicide
- cervical sponges
- condoms with spermicide

**Sotrovimab-specific eligibility criteria**

In addition to the inclusion and exclusion criteria outlined in the master protocol, the following patients will be excluded: 1) pregnant women; and 2) nursing mothers. In addition, prior to the initial futility assessment which is performed when approximately 150 participants have been enrolled on VIR-7831 and 150 on placebo, patients on high-flow oxygen or non-invasive ventilation (category 5 of the pulmonary ordinal outcome) will be excluded. These patients may be eligible for the trial if the initial futility assessment is passed by this agent.

**Amubarvimab–romlusevimab specific eligibility criteria**

In addition to the inclusion and exclusion criteria outlined in the master protocol, the following patients will be excluded: 1) pregnant women; and 2) nursing mothers. In addition, prior to the initial futility assessment which is performed when approximately 150 participants have been enrolled on BRII-196/BRII-198 and 150 on placebo, patients on high-flow oxygen or non-invasive ventilation (category 5 of the pulmonary ordinal outcome) will be excluded. These patients may be eligible for the trial if the initial futility assessment is passed by this agent.

**Tixagevimab-cilgavimab specific eligibility criteria**

In addition to the inclusion and exclusion criteria outlined in the master protocol, the following patients will be excluded: 1) pregnant women; and 2) nursing mothers. In addition, prior to the initial futility assessment which is performed when approximately 150 participants have been enrolled on AZD7442 and 150 on placebo, patients on high-flow oxygen or non-invasive ventilation (category 5 of the pulmonary ordinal outcome) will be excluded. These patients may be eligible for the trial if the initial futility assessment is passed by this agent.

MP-specific eligibility criteria

Exclude pregnant and breastfeeding women. Otherwise, eligibility criteria as outlined in the master protocol are used for the study of this agent.

Not all antiviral products being evaluated were available throughout the 16-month enrollment period, therefore relatively few participants were eligible to be randomized to more than one antiviral product at the same time. Most participants were randomized 1:1 with placebo. Background standard of care mandated remdesivir unless contraindicated, and steroids were utilized based on hospital-specific guidelines.

## Clinical Data

Prior to randomization, case report forms common across antiviral products collected data for each TICO trial participant including demographic characteristics (age, sex, and race/ethnicity), geographic location (United States, Europe, Asia, Africa), residence before becoming ill with COVID-19 (independent dwelling without professional medical help vs. any other residence), infection period (pre-2021, January-June 2021, July-November 2021), pre-COVID comorbidities defined as active or receiving treatment for in the 12 months prior to randomization (asthma, COPD, cerebrovascular event, diabetes, heart failure, hepatic impairment, HIV, immunosuppressive disorder other than HIV, hypertension, malignancy, myocardial infarction or other acute coronary syndrome, renal impairment, body mass index [BMI]), SARS-CoV-2 vaccination status (0, 1 or 2 doses received at least 14 days prior), number of days since COVID-19 symptom onset, concomitant medications taken in the 24 hours prior to randomization (antibacterials, antifungals, angiotensin-converting-enzyme (ACE) inhibitors, angiotensin receptor blockers (ARBs), antiplatelet/anticoagulant medications, antivirals against SARS-CoV-2, antirejection medicine, immune modulators, Non-steroidal anti-inflammatory drugs (NSAIDs), corticosteroids, and treatment with biological medicine to treat autoimmune disease or cancer), pulmonary status defined by respiratory support needs (categories: no supplemental oxygen (O_2_), O_2_ <4 L/min, O_2_ ≥4 L/min, high-flow nasal cannula [HFNC] or noninvasive ventilation [NIV]), clinical severity metrics including the modified Borg dyspnea scale, and National Early Warning Score (NEWS).

At the day (D) 5 visit, concomitant medications taken in the past 24 hours and local clinical labs that were collected. The number of doses of remdesivir received and the dates of the first and last doses were recorded.

## Local Laboratory Measurements

Local laboratory is defined as the clinical laboratory at or associated with the hospital where the participant was hospitalized. Absolute lymphocyte count (ALC), serum creatinine (Cr), aspartate aminotransferase/serum glutamic-oxaloacetic transaminase (AST/SGOT), alanine aminotransferase/ serum glutamic-pyruvic transaminase (ALT/SGPT), white blood cell (WBC), hemoglobin, and platelets were collected locally and reported on electronic case report forms. These clinical laboratory measurements were collected on D0 (within 24 hours prior to randomization) and at D5 (+/- 1 day was allowed if collection on D5 was not possible).

## Central Laboratory Measurements

Central laboratory is defined as the NIAID funded laboratories at the Frederick National Laboratory for Cancer Research/Leidos Biomedical Research, Inc. in Frederick, MD. The central laboratories are CLIA-certified. Banked plasma collected at D0 (within 24 hours prior to randomization) and D5 (+/- 1 day was allowed if collection on D5 was not possible) was run centrally using the quantitative SARS-CoV-2 N antigen microbead-based immunoassay (Quanterix); the lower limit of quantification is 3 ng/L. Elevated levels of plasma N antigen have previously been shown to be associated with poor pulmonary outcomes, time to hospital discharge, and mortality(1,2). SARS-CoV-2 viral RNA was extracted centrally from a mid-turbinate nasal swab collected at day 0. Viral load was determined using an RT-PCR assay with a standard curve of known concentrations as described previously (2). Antibody measurements were done centrally on the days 0 and 5 plasma. Anti-spike neutralizing antibody was determined using a surrogate viral neutralization test (GenScript cPass); results of 30% or more were considered positive. Anti-N pan-Ig positivity was determined according to the manufacturer's directions (Bio-Rad Platelia SARS-CoV-2 Total Antibody Test). Interleukin 6 (IL-6), C-reactive protein (CRP), and D-dimer were measured centrally on stored plasma from days 0 and 5. Plasma levels of IL-6 and CRP were measured using electrochemiluminescence (Meso Scale Discovery, Gaithersburg, MD).  D-dimer was measured by an enzyme-linked fluorescent assay on a VIDAS instrument (BioMerieux, Durham, NC).  Each assay was run according to the manufacturer’s instructions included in the kit. In cases where CRP levels reached the upper limit of the assay, a 1:10,000 fold dilution was used.

## Statistical Analysis

Construction of categorical variables from continuously measured variables: Lymphopenia was defined using a clinically relevant cutoff of ALC (< 0.9 x10^9^/L) and severe lymphopenia was defined using the lowest ALC quartile of our population at Day 0 (< 0.56 x10^9^/L). BMI, CRP, and estimated glomerular filtration rate (eGFR) were reported using clinically relevant, commonly used cutoffs, whereas other variables were made categorical by using observed quantiles (e.g., viral load, IL-6, D-dimer). Plasma viral Ag was dichotomized using a previously published cutoff (1) but was subject to further categorization using quartiles to better understand the impact of this important variable. The modified Borg scale was collapsed into 3 categories based on the interpretation of the scale (nothing to slight, severe or worse, and intermediate). Similarly, the categories of the NEWS score were collapsed to merge adjacent categories.

Missing data: Baseline centrally run laboratory measurements are not available for some participants. CRP is missing for 265, IL-6 and D-dimer are missing for 145, SARS-CoV-2 viral load is missing for 107, and plasma Ag is missing for 77. In baseline summary tables, percentages are calculated among those who have available data. In adjusted models, complete-case analyses were performed which resulted in reduced sample sizes. In adjusted models examining Day 0 lymphopenia, 2236 out of 2579 (87%) had complete data and were included in the analyses. The adjusted lymphopenia trajectory models include 1861 out of 2105 (88%) with complete data. New lymphopenia compared to no lymphopenia models include 817 (89%) participants and resolved lymphopenia compared to persistent lymphopenia models include 1039 (88%) participants.

# Section 2: Supplemental Tables and Figures

## Figure E1. Flowchart of Participant Selection

**D0 and D5 ALC available (n=2105)**

**ALC trajectory analysis population**

D5 ALC not available (n=474)

D0 ALC not available (n=46)

Did not receive any of the assigned study product (n=128)

**D0 ALC available (n=2579)**

**Analysis population**

Received all or part of assigned study product (n=2625)

TICO mITT population

Randomized to receive one of 5 antiviral products in TICO (n=2753)

## Table E1. Baseline characteristics by Day 0 Lymphopenia Status

|  | **ALC < 0.9**  **(N=1426)** | | **ALC ≥ 0.9**  **(N=1153)** | | **Total**  **(N=2579)** | |
| --- | --- | --- | --- | --- | --- | --- |
|  | **N** | **(%)** | **N** | **(%)** | **N** | **(%)** |
| **Age - med. (IQR) years** | 59 (48 - 70) | | 55 (44 - 66) | | 57 (46 - 68) | |
| 18-39 years | 155 | 10.9 | 197 | 17.1 | 352 | 13.6 |
| 40-49 years | 238 | 16.7 | 238 | 20.6 | 476 | 18.5 |
| 50-59 years | 346 | 24.3 | 272 | 23.6 | 618 | 24.0 |
| 60-69 years | 326 | 22.9 | 233 | 20.2 | 559 | 21.7 |
| 70-79 years | 245 | 17.2 | 154 | 13.4 | 399 | 15.5 |
| ≥ 80 years | 116 | 8.1 | 59 | 5.1 | 175 | 6.8 |
| **Sex** |  |  |  |  |  |  |
| Male | 882 | 61.9 | 602 | 52.2 | 1484 | 57.5 |
| Female | 544 | 38.1 | 551 | 47.8 | 1095 | 42.5 |
| **Race/ethnicity** |  |  |  |  |  |  |
| Asian | 73 | 5.1 | 46 | 4.0 | 119 | 4.6 |
| Black | 317 | 22.2 | 302 | 26.2 | 619 | 24.0 |
| Hispanic | 242 | 17.0 | 232 | 20.1 | 474 | 18.4 |
| White | 752 | 52.7 | 528 | 45.8 | 1280 | 49.6 |
| Other | 42 | 2.9 | 45 | 3.9 | 87 | 3.4 |
| **Region** |  |  |  |  |  |  |
| United States | 1169 | 82.0 | 850 | 73.7 | 2019 | 78.3 |
| Europe | 191 | 13.4 | 198 | 17.2 | 389 | 15.1 |
| Africa | 40 | 2.8 | 89 | 7.7 | 129 | 5.0 |
| Asia | 26 | 1.8 | 16 | 1.4 | 42 | 1.6 |
| **Residence** |  |  |  |  |  |  |
| Independent, w/o assistance | 1330 | 93.3 | 1101 | 95.5 | 2431 | 94.3 |
| Other | 96 | 6.7 | 52 | 4.5 | 148 | 5.7 |
| **Date of infection** |  |  |  |  |  |  |
| Pre 2021 | 241 | 16.9 | 160 | 13.9 | 401 | 15.5 |
| Jan-Jun 2021 | 565 | 39.6 | 469 | 40.7 | 1034 | 40.1 |
| Jul-Dec 2021 | 620 | 43.5 | 524 | 45.4 | 1144 | 44.4 |
| **Symptom duration - med. (IQR) days** | 8 (6 - 10) | | 8 (6 - 10) | | 8 (6 - 10) | |
| < 5 | 208 | 14.6 | 185 | 16.0 | 393 | 15.2 |
| 5 - 7 | 414 | 29.0 | 335 | 29.1 | 749 | 29.0 |
| 8 - 10 | 595 | 41.7 | 475 | 41.2 | 1070 | 41.5 |
| > 10 | 209 | 14.7 | 158 | 13.7 | 367 | 14.2 |
| **# vaccine doses** |  |  |  |  |  |  |
| 0 | 1158 | 81.8 | 956 | 83.7 | 2114 | 82.7 |
| 1 | 105 | 7.4 | 74 | 6.5 | 179 | 7.0 |
| 2 | 152 | 10.7 | 112 | 9.8 | 264 | 10.3 |
| **Quanterix Ag - med. (IQR) ng/L** | 2093 (466 - 5845) | | 847 (97 - 3232) | | 1448 (235 - 4753) | |
| 1000+ | 905 | 65.5 | 529 | 47.2 | 1434 | 57.3 |
| < 1000 | 477 | 34.5 | 591 | 52.8 | 1068 | 42.7 |
| **SARS-CoV-2 viral load** |  |  |  |  |  |  |
| Negative | 156 | 11.3 | 173 | 15.8 | 329 | 13.3 |
| < 35,000 copies/mL | 577 | 41.9 | 493 | 45.0 | 1070 | 43.3 |
| 35,000+ copies/mL | 643 | 46.7 | 430 | 39.2 | 1073 | 43.4 |
| **Anti-spike Ab** |  |  |  |  |  |  |
| Positive | 677 | 49.0 | 612 | 54.6 | 1289 | 51.5 |
| Negative | 705 | 51.0 | 508 | 45.4 | 1213 | 48.5 |
| **Anti-N Ab** |  |  |  |  |  |  |
| Positive | 845 | 61.1 | 714 | 63.7 | 1559 | 62.3 |
| Negative | 537 | 38.9 | 407 | 36.3 | 944 | 37.7 |
| **Asthma** |  |  |  |  |  |  |
| Yes | 129 | 9.0 | 126 | 10.9 | 255 | 9.9 |
| No | 1297 | 91.0 | 1027 | 89.1 | 2324 | 90.1 |
| **COPD** |  |  |  |  |  |  |
| Yes | 86 | 6.0 | 75 | 6.5 | 161 | 6.2 |
| No | 1340 | 94.0 | 1078 | 93.5 | 2418 | 93.8 |
| **Diabetes** |  |  |  |  |  |  |
| Yes | 421 | 29.5 | 305 | 26.5 | 726 | 28.2 |
| No | 1005 | 70.5 | 848 | 73.5 | 1853 | 71.8 |
| **Heart failure** |  |  |  |  |  |  |
| Yes | 75 | 5.3 | 39 | 3.4 | 114 | 4.4 |
| No | 1351 | 94.7 | 1114 | 96.6 | 2465 | 95.6 |
| **Hypertension** |  |  |  |  |  |  |
| Yes | 703 | 49.3 | 475 | 41.2 | 1178 | 45.7 |
| No | 723 | 50.7 | 678 | 58.8 | 1401 | 54.3 |
| **Renal impairment** |  |  |  |  |  |  |
| Yes | 188 | 13.2 | 69 | 6.0 | 257 | 10.0 |
| No | 1238 | 86.8 | 1084 | 94.0 | 2322 | 90.0 |
| **BMI - med. (IQR)** | 30 (26 - 35) | | 31 (27 - 37) | | 30 (26 - 36) | |
| < 18.5 (underweight) | 22 | 1.5 | 25 | 2.2 | 47 | 1.8 |
| 18.5-24.9 (healthy) | 258 | 18.1 | 164 | 14.3 | 422 | 16.4 |
| 25-29.9 (overweight) | 428 | 30.1 | 315 | 27.4 | 743 | 28.9 |
| 30-39.9 (obese) | 532 | 37.4 | 453 | 39.4 | 985 | 38.3 |
| ≥ 40 (morbidly obese) | 182 | 12.8 | 192 | 16.7 | 374 | 14.5 |
| **Immunomodulators** |  |  |  |  |  |  |
| Yes | 106 | 7.4 | 62 | 5.4 | 168 | 6.5 |
| No | 1320 | 92.6 | 1091 | 94.6 | 2411 | 93.5 |
| **Corticosteroids** |  |  |  |  |  |  |
| Yes | 1040 | 72.9 | 716 | 62.1 | 1756 | 68.1 |
| No | 386 | 27.1 | 437 | 37.9 | 823 | 31.9 |
| **Remdesivir prior to rand.** |  |  |  |  |  |  |
| Yes | 895 | 62.8 | 679 | 58.9 | 1574 | 61.0 |
| No | 531 | 37.2 | 474 | 41.1 | 1005 | 39.0 |
| **Pulmonary status** |  |  |  |  |  |  |
| No O2 | 307 | 21.5 | 337 | 29.2 | 644 | 25.0 |
| O2 < 4 L/min | 512 | 35.9 | 420 | 36.4 | 932 | 36.1 |
| O2 ≥ 4 L/min | 424 | 29.7 | 295 | 25.6 | 719 | 27.9 |
| Non-invasive vent./HFNC | 183 | 12.8 | 101 | 8.8 | 284 | 11.0 |
| **Borg Dyspnea Scale** |  |  |  |  |  |  |
| 0-2 (nothing to slight) | 618 | 46.9 | 515 | 48.8 | 1133 | 47.7 |
| 3-4 (mod-somewhat severe) | 413 | 31.3 | 360 | 34.1 | 773 | 32.6 |
| 5-10 (severe-maximal) | 287 | 21.8 | 180 | 17.1 | 467 | 19.7 |
| **NEWS** |  |  |  |  |  |  |
| < 2 | 147 | 10.4 | 164 | 14.3 | 311 | 12.1 |
| 2-3 | 441 | 31.1 | 397 | 34.5 | 838 | 32.6 |
| 4-5 | 466 | 32.9 | 355 | 30.9 | 821 | 32.0 |
| ≥ 6 | 363 | 25.6 | 234 | 20.3 | 597 | 23.3 |
| **Platelets – med. (IQR) x10^9^/L** | 198 (155 - 251) | | 223 (177 - 292) | | 209 (163 - 267) | |
| **Hemoglobin – med. (IQR) g/dL** | 13.1 (11.7 - 14.3) | | 13.3 (12.1 - 14.4) | | 13.2 (11.9 - 14.3) | |
| **Serum creatinine - med. (IQR) mg/dL** | 0.90 (0.71 - 1.16) | | 0.81 (0.68 - 1.01) | | 0.86 (0.70 - 1.10) | |
| < 1.1 | 989 | 69.5 | 913 | 79.3 | 1902 | 73.8 |
| 1.1-1.5 | 251 | 17.6 | 149 | 12.9 | 400 | 15.5 |
| > 1.5 | 184 | 12.9 | 90 | 7.8 | 274 | 10.6 |
| **eGFR - med. (IQR)** | 88 (62 - 105) | | 95 (75 - 111) | | 91 (68 - 107) | |
| < 60 | 332 | 23.3 | 165 | 14.3 | 497 | 19.3 |
| ≥ 60 | 1092 | 76.7 | 987 | 85.7 | 2079 | 80.7 |
| **CRP - med. (IQR) mg/L** | 77 (36 - 134) | | 50 (20 - 95) | | 62 (28 - 118) | |
| < 5 | 477 | 33.9 | 567 | 49.9 | 1044 | 41.0 |
| 5-7.5 | 217 | 15.4 | 192 | 16.9 | 409 | 16.1 |
| > 7.5 | 715 | 50.7 | 378 | 33.2 | 1093 | 42.9 |
| **IL-6 - med (IQR) ng/L** | 6 (2 - 15) | | 6 (2 - 14) | | 6 (2 - 15) | |
| ≤ 5.8 | 650 | 48.4 | 557 | 51.1 | 1207 | 49.6 |
| > 5.8 | 694 | 51.6 | 533 | 48.9 | 1227 | 50.4 |
| **D-dimer – med (IQR) mg/L** | 1.00 (0.68 - 1.57) | | 0.85 (0.57 - 1.37) | | 0.93 (0.63 - 1.47) | |
| ≤ 0.93 | 602 | 44.8 | 611 | 56.1 | 1213 | 49.8 |
| > 0.93 | 742 | 55.2 | 479 | 43.9 | 1221 | 50.2 |
|  | | | | | | |

IQR=interquartile range, Ag=antigen, Ab=antibody, COPD=chronic obstructive pulmonary disease, BMI=body mass index, HFNC=high flow nasal canula, NEWS=National Early Warning Score, eGFR=estimated glomerular filtration rate, CRP=C-reactive protein, IL-6=interleukin 6

Percentages are calculated among those with available data for each variable

## Table E2. Baseline characteristics by Day 0 Severe Lymphopenia Status

|  | **ALC < 0.56**  **(N=636)** | | **ALC ≥ 0.56**  **(N=1943)** | | **Total**  **(N=2579)** | |
| --- | --- | --- | --- | --- | --- | --- |
|  | **N** | **(%)** | **N** | **(%)** | **N** | **(%)** |
| **Age - med. (IQR) years** | 61 (50 - 71) | | 56 (44 - 67) | | 57 (46 - 68) | |
| 18-39 years | 51 | 8.0 | 301 | 15.5 | 352 | 13.6 |
| 40-49 years | 91 | 14.3 | 385 | 19.8 | 476 | 18.5 |
| 50-59 years | 148 | 23.3 | 470 | 24.2 | 618 | 24.0 |
| 60-69 years | 169 | 26.6 | 390 | 20.1 | 559 | 21.7 |
| 70-79 years | 120 | 18.9 | 279 | 14.4 | 399 | 15.5 |
| ≥ 80 years | 57 | 9.0 | 118 | 6.1 | 175 | 6.8 |
| **Sex** |  |  |  |  |  |  |
| Male | 387 | 60.8 | 1097 | 56.5 | 1484 | 57.5 |
| Female | 249 | 39.2 | 846 | 43.5 | 1095 | 42.5 |
| **Race/ethnicity** |  |  |  |  |  |  |
| Asian | 24 | 3.8 | 95 | 4.9 | 119 | 4.6 |
| Black | 148 | 23.3 | 471 | 24.2 | 619 | 24.0 |
| Hispanic | 97 | 15.3 | 377 | 19.4 | 474 | 18.4 |
| White | 350 | 55.0 | 930 | 47.9 | 1280 | 49.6 |
| Other | 17 | 2.7 | 70 | 3.6 | 87 | 3.4 |
| **Region** |  |  |  |  |  |  |
| United States | 550 | 86.5 | 1469 | 75.6 | 2019 | 78.3 |
| Europe | 63 | 9.9 | 326 | 16.8 | 389 | 15.1 |
| Africa | 16 | 2.5 | 113 | 5.8 | 129 | 5.0 |
| Asia | 7 | 1.1 | 35 | 1.8 | 42 | 1.6 |
| **Residence** |  |  |  |  |  |  |
| Independent, w/o assistance | 588 | 92.5 | 1843 | 94.9 | 2431 | 94.3 |
| Other | 48 | 7.5 | 100 | 5.1 | 148 | 5.7 |
| **Date of infection** |  |  |  |  |  |  |
| Pre 2021 | 107 | 16.8 | 294 | 15.1 | 401 | 15.5 |
| Jan-Jun 2021 | 244 | 38.4 | 790 | 40.7 | 1034 | 40.1 |
| Jul-Dec 2021 | 285 | 44.8 | 859 | 44.2 | 1144 | 44.4 |
| **Symptom duration - med. (IQR) days** | 8 (6 - 9) | | 8 (6 - 10) | | 8 (6 - 10) | |
| < 5 | 103 | 16.2 | 290 | 14.9 | 393 | 15.2 |
| 5 - 7 | 195 | 30.7 | 554 | 28.5 | 749 | 29.0 |
| 8 - 10 | 247 | 38.8 | 823 | 42.4 | 1070 | 41.5 |
| > 10 | 91 | 14.3 | 276 | 14.2 | 367 | 14.2 |
| **# vaccine doses** |  |  |  |  |  |  |
| 0 | 504 | 79.6 | 1610 | 83.7 | 2114 | 82.7 |
| 1 | 43 | 6.8 | 136 | 7.1 | 179 | 7.0 |
| 2 | 86 | 13.6 | 178 | 9.3 | 264 | 10.3 |
| **Quanterix Ag - med. (IQR) ng/L** | 2724 (587 - 6779) | | 1199 (168 - 4000) | | 1448 (235 - 4753) | |
| 1000+ | 425 | 68.7 | 1009 | 53.6 | 1434 | 57.3 |
| < 1000 | 194 | 31.3 | 874 | 46.4 | 1068 | 42.7 |
| **SARS-CoV-2 viral load** |  |  |  |  |  |  |
| Negative | 59 | 9.6 | 270 | 14.5 | 329 | 13.3 |
| < 35,000 copies/mL | 233 | 37.8 | 837 | 45.1 | 1070 | 43.3 |
| 35,000+ copies/mL | 324 | 52.6 | 749 | 40.4 | 1073 | 43.4 |
| **Anti-spike Ab** |  |  |  |  |  |  |
| Positive | 281 | 45.4 | 1008 | 53.5 | 1289 | 51.5 |
| Negative | 338 | 54.6 | 875 | 46.5 | 1213 | 48.5 |
| **Anti-N Ab** |  |  |  |  |  |  |
| Positive | 362 | 58.5 | 1197 | 63.5 | 1559 | 62.3 |
| Negative | 257 | 41.5 | 687 | 36.5 | 944 | 37.7 |
| **Asthma** |  |  |  |  |  |  |
| Yes | 45 | 7.1 | 210 | 10.8 | 255 | 9.9 |
| No | 591 | 92.9 | 1733 | 89.2 | 2324 | 90.1 |
| **COPD** |  |  |  |  |  |  |
| Yes | 47 | 7.4 | 114 | 5.9 | 161 | 6.2 |
| No | 589 | 92.6 | 1829 | 94.1 | 2418 | 93.8 |
| **Diabetes** |  |  |  |  |  |  |
| Yes | 190 | 29.9 | 536 | 27.6 | 726 | 28.2 |
| No | 446 | 70.1 | 1407 | 72.4 | 1853 | 71.8 |
| **Heart failure** |  |  |  |  |  |  |
| Yes | 44 | 6.9 | 70 | 3.6 | 114 | 4.4 |
| No | 592 | 93.1 | 1873 | 96.4 | 2465 | 95.6 |
| **Hypertension** |  |  |  |  |  |  |
| Yes | 327 | 51.4 | 851 | 43.8 | 1178 | 45.7 |
| No | 309 | 48.6 | 1092 | 56.2 | 1401 | 54.3 |
| **Renal impairment** |  |  |  |  |  |  |
| Yes | 108 | 17.0 | 149 | 7.7 | 257 | 10.0 |
| No | 528 | 83.0 | 1794 | 92.3 | 2322 | 90.0 |
| **BMI - med. (IQR)** | 29 (26 - 35) | | 31 (27 - 36) | | 30 (26 - 36) | |
| < 18.5 (underweight) | 13 | 2.1 | 34 | 1.8 | 47 | 1.8 |
| 18.5-24.9 (healthy) | 128 | 20.2 | 294 | 15.2 | 422 | 16.4 |
| 25-29.9 (overweight) | 196 | 31.0 | 547 | 28.2 | 743 | 28.9 |
| 30-39.9 (obese) | 229 | 36.2 | 756 | 39.0 | 985 | 38.3 |
| ≥ 40 (morbidly obese) | 67 | 10.6 | 307 | 15.8 | 374 | 14.5 |
| **Immunomodulators** |  |  |  |  |  |  |
| Yes | 54 | 8.5 | 114 | 5.9 | 168 | 6.5 |
| No | 582 | 91.5 | 1829 | 94.1 | 2411 | 93.5 |
| **Corticosteroids** |  |  |  |  |  |  |
| Yes | 480 | 75.5 | 1276 | 65.7 | 1756 | 68.1 |
| No | 156 | 24.5 | 667 | 34.3 | 823 | 31.9 |
| **Remdesivir prior to rand.** |  |  |  |  |  |  |
| Yes | 408 | 64.2 | 1166 | 60.0 | 1574 | 61.0 |
| No | 228 | 35.8 | 777 | 40.0 | 1005 | 39.0 |
| **Pulmonary status** |  |  |  |  |  |  |
| No O2 | 124 | 19.5 | 520 | 26.8 | 644 | 25.0 |
| O2 < 4 L/min | 216 | 34.0 | 716 | 36.9 | 932 | 36.1 |
| O2 ≥ 4 L/min | 208 | 32.7 | 511 | 26.3 | 719 | 27.9 |
| Non-invasive vent./HFNC | 88 | 13.8 | 196 | 10.1 | 284 | 11.0 |
| **Borg Dyspnea Scale** |  |  |  |  |  |  |
| 0-2 (nothing to slight) | 281 | 47.4 | 852 | 47.9 | 1133 | 47.7 |
| 3-4 (mod-somewhat severe) | 178 | 30.0 | 595 | 33.4 | 773 | 32.6 |
| 5-10 (severe-maximal) | 134 | 22.6 | 333 | 18.7 | 467 | 19.7 |
| **NEWS** |  |  |  |  |  |  |
| < 2 | 62 | 9.7 | 249 | 12.9 | 311 | 12.1 |
| 2-3 | 184 | 28.9 | 654 | 33.9 | 838 | 32.6 |
| 4-5 | 212 | 33.3 | 609 | 31.5 | 821 | 32.0 |
| ≥ 6 | 178 | 28.0 | 419 | 21.7 | 597 | 23.3 |
| **Platelets – med. (IQR) x10^9^/L** | 190 (141 - 243) | | 215 (169 - 247) | | 209 (163 - 267) | |
| **Hemoglobin – med. (IQR) g/dL** | 12.7 (11.2 -14.1) | | 13.3 (12.1 - 14.4) | | 13.2 (11.9 - 14.3) | |
| **Serum creatinine - med. (IQR) mg/dL** | 0.90 (0.72 - 1.24) | | 0.85 (0.70 - 1.06) | | 0.86 (0.70 - 1.10) | |
| < 1.1 | 413 | 65.0 | 1489 | 76.7 | 1902 | 73.8 |
| 1.1-1.5 | 111 | 17.5 | 289 | 14.9 | 400 | 15.5 |
| > 1.5 | 111 | 17.5 | 163 | 8.4 | 274 | 10.6 |
| **eGFR - med. (IQR)** | 83 (55 - 102) | | 93 (71 - 109) | | 91 (68 - 107) | |
| < 60 | 180 | 28.3 | 317 | 16.3 | 497 | 19.3 |
| ≥ 60 | 455 | 71.7 | 1624 | 83.7 | 2079 | 80.7 |
| **CRP - med. (IQR) mg/L** | 84 (42 - 145) | | 57 (25 - 109) | | 62 (28 - 118) | |
| < 5 | 190 | 30.4 | 854 | 44.5 | 1044 | 41.0 |
| 5-7.5 | 92 | 14.7 | 317 | 16.5 | 409 | 16.1 |
| > 7.5 | 344 | 55.0 | 749 | 39.0 | 1093 | 42.9 |
| **IL-6 - med (IQR) ng/L** | 7 (3 - 18) | | 6 (2 - 13) | | 6 (2 - 15) | |
| ≤ 5.8 | 267 | 44.3 | 940 | 51.3 | 1207 | 49.6 |
| > 5.8 | 336 | 55.7 | 891 | 48.7 | 1227 | 50.4 |
| **D-dimer – med (IQR) mg/L** | 1.11 (0.76 - 1.81) | | 0.88 (0.60 -1.39) | | 0.93 (0.63 - 1.47) | |
| ≤ 0.93 | 227 | 37.6 | 986 | 53.9 | 1213 | 49.8 |
| > 0.93 | 376 | 62.4 | 845 | 46.1 | 1221 | 50.2 |

IQR=interquartile range, Ag=antigen, Ab=antibody, COPD=chronic obstructive pulmonary disease, BMI=body mass index, HFNC=high flow nasal canula, NEWS=National Early Warning Score, eGFR=estimated glomerular filtration rate, CRP=C-reactive protein, IL-6=interleukin 6

Percentages are calculated among those with available data for each variable

## Table E3. Reasons for Missing Day 5 ALC

|  | **N** | **%** |
| --- | --- | --- |
| **No Day 5 visit** |  |  |
| Died by day 5 | 25 | 5.3 |
| Withdrew consent by day 5 | 14 | 3.0 |
| Lost to follow-up by day 5 | 2 | 0.4 |
| Missed visit | 229 | 48.3 |
| **Day 5 visit attended** |  |  |
| Other local labs collected | 91 | 19.2 |
| Central labs collected, no local labs | 60 | 12.7 |
| No labs collected | 53 | 11.2 |
| **Total** | 474 | 100.0 |
|  | | |

## Table E4. Baseline characteristics by Severe Lymphopenia (< 0.56) Trajectory

|  | **No Severe Lymphopenia (n= 1487)** | | **Resolved Severe Lymphopenia  (n= 351)** | | **New Severe Lymphopenia (n= 93)** | | **Persistent Severe Lymphopenia (n= 174)** | |
| --- | --- | --- | --- | --- | --- | --- | --- | --- |
|  | **N** | **(%)** | **N** | **(%)** | **N** | **(%)** | **N** | **(%)** |
| **Age - med. (IQR) years** | 55 (44 - 66) | | 58 (50 - 68) | | 66 (56 - 75) | | 65 (55 - 73) | |
| 18-39 years | 230 | 15.5 | 33 | 9.4 | 5 | 5.4 | 9 | 5.2 |
| 40-49 years | 311 | 20.9 | 53 | 15.1 | 8 | 8.6 | 18 | 10.3 |
| 50-59 years | 370 | 24.9 | 98 | 27.9 | 18 | 19.4 | 30 | 17.2 |
| 60-69 years | 302 | 20.3 | 89 | 25.4 | 26 | 28.0 | 59 | 33.9 |
| 70-79 years | 194 | 13.0 | 61 | 17.4 | 26 | 28.0 | 35 | 20.1 |
| ≥ 80 years | 80 | 5.4 | 17 | 4.8 | 10 | 10.8 | 23 | 13.2 |
| **Sex** |  |  |  |  |  |  |  |  |
| Male | 825 | 55.5 | 205 | 58.4 | 68 | 73.1 | 114 | 65.5 |
| Female | 662 | 44.5 | 146 | 41.6 | 25 | 26.9 | 60 | 34.5 |
| **Race/ethnicity** |  |  |  |  |  |  |  |  |
| Asian | 75 | 5.0 | 15 | 4.3 | 9 | 9.7 | 7 | 4.0 |
| Black | 368 | 24.7 | 83 | 23.6 | 7 | 7.5 | 40 | 23.0 |
| Hispanic | 289 | 19.4 | 55 | 15.7 | 17 | 18.3 | 27 | 15.5 |
| White | 702 | 47.2 | 190 | 54.1 | 56 | 60.2 | 95 | 54.6 |
| Other | 53 | 3.6 | 8 | 2.3 | 4 | 4.3 | 5 | 2.9 |
| **Region** |  |  |  |  |  |  |  |  |
| United States | 1064 | 71.6 | 296 | 84.3 | 76 | 81.7 | 152 | 87.4 |
| Europe | 291 | 19.6 | 43 | 12.3 | 11 | 11.8 | 14 | 8.0 |
| Africa | 103 | 6.9 | 9 | 2.6 | 2 | 2.2 | 4 | 2.3 |
| Asia | 29 | 2.0 | 3 | 0.9 | 4 | 4.3 | 4 | 2.3 |
| **Residence** |  |  |  |  |  |  |  |  |
| Independent, w/o assistance | 1409 | 94.8 | 326 | 92.9 | 88 | 94.6 | 163 | 93.7 |
| Other | 78 | 5.2 | 25 | 7.1 | 5 | 5.4 | 11 | 6.3 |
| **Date of infection** |  |  |  |  |  |  |  |  |
| Pre 2021 | 214 | 14.4 | 63 | 17.9 | 17 | 18.3 | 27 | 15.5 |
| Jan-Jun 2021 | 601 | 40.4 | 141 | 40.2 | 31 | 33.3 | 63 | 36.2 |
| Jul-Dec 2021 | 672 | 45.2 | 147 | 41.9 | 45 | 48.4 | 84 | 48.3 |
| **Symptom duration - med. (IQR) days** | 8 (6 - 10) | | 8 (6 - 10) | | 7 (5 - 10) | | 8 (5 - 9) | |
| < 5 | 215 | 14.5 | 49 | 14.0 | 18 | 19.4 | 32 | 18.4 |
| 5 - 7 | 413 | 27.8 | 106 | 30.2 | 30 | 32.3 | 52 | 29.9 |
| 8 - 10 | 641 | 43.1 | 141 | 40.2 | 33 | 35.5 | 64 | 36.8 |
| > 10 | 218 | 14.7 | 55 | 15.7 | 12 | 12.9 | 26 | 14.9 |
| **# vaccine doses** |  |  |  |  |  |  |  |  |
| 0 | 1234 | 83.6 | 296 | 85.1 | 72 | 80.9 | 125 | 71.8 |
| 1 | 106 | 7.2 | 16 | 4.6 | 5 | 5.6 | 18 | 10.3 |
| 2 | 136 | 9.2 | 36 | 10.3 | 12 | 13.5 | 31 | 17.8 |
| **Quanterix Ag - med. (IQR) ng/L** | 1134 (173 - 4003) | | 2452 (540 - 6130) | | 3107 (1232 - 8431) | | 4629 (1180 - 10911) | |
| 1000+ | 764 | 52.5 | 233 | 67.0 | 68 | 76.4 | 126 | 76.8 |
| < 1000 | 692 | 47.5 | 115 | 33.0 | 21 | 23.6 | 38 | 23.2 |
| **SARS-CoV-2 viral load** |  |  |  |  |  |  |  |  |
| Negative | 209 | 14.5 | 36 | 10.6 | 6 | 6.5 | 13 | 7.7 |
| < 35,000 copies/mL | 652 | 45.3 | 145 | 42.5 | 41 | 44.6 | 47 | 28.0 |
| 35,000+ copies/mL | 578 | 40.2 | 160 | 46.9 | 45 | 48.9 | 108 | 64.3 |
| **Anti-spike Ab** |  |  |  |  |  |  |  |  |
| Positive | 778 | 53.4 | 170 | 48.9 | 42 | 47.2 | 58 | 35.4 |
| Negative | 678 | 46.6 | 178 | 51.1 | 47 | 52.8 | 106 | 64.6 |
| **Anti-N Ab** |  |  |  |  |  |  |  |  |
| Positive | 933 | 64.0 | 218 | 62.6 | 51 | 57.3 | 81 | 49.4 |
| Negative | 524 | 36.0 | 130 | 37.4 | 38 | 42.7 | 83 | 50.6 |
| **Asthma** |  |  |  |  |  |  |  |  |
| Yes | 151 | 10.2 | 24 | 6.8 | 8 | 8.6 | 13 | 7.5 |
| No | 1336 | 89.8 | 327 | 93.2 | 85 | 91.4 | 161 | 92.5 |
| **COPD** |  |  |  |  |  |  |  |  |
| Yes | 82 | 5.5 | 20 | 5.7 | 7 | 7.5 | 17 | 9.8 |
| No | 1405 | 94.5 | 331 | 94.3 | 86 | 92.5 | 157 | 90.2 |
| **Diabetes** |  |  |  |  |  |  |  |  |
| Yes | 396 | 26.6 | 106 | 30.2 | 29 | 31.2 | 50 | 28.7 |
| No | 1091 | 73.4 | 245 | 69.8 | 64 | 68.8 | 124 | 71.3 |
| **Heart failure** |  |  |  |  |  |  |  |  |
| Yes | 50 | 3.4 | 14 | 4.0 | 5 | 5.4 | 20 | 11.5 |
| No | 1437 | 96.6 | 337 | 96.0 | 88 | 94.6 | 154 | 88.5 |
| **Hypertension** |  |  |  |  |  |  |  |  |
| Yes | 633 | 42.6 | 173 | 49.3 | 50 | 53.8 | 94 | 54.0 |
| No | 854 | 57.4 | 178 | 50.7 | 43 | 46.2 | 80 | 46.0 |
| **Renal impairment** |  |  |  |  |  |  |  |  |
| Yes | 111 | 7.5 | 42 | 12.0 | 13 | 14.0 | 46 | 26.4 |
| No | 1376 | 92.5 | 309 | 88.0 | 80 | 86.0 | 128 | 73.6 |
| **BMI - med. (IQR)** | 31 (27 - 36) | | 30 (26 - 35) | | 28 (25 - 33) | | 29 (25 - 34) | |
| < 18.5 (underweight) | 29 | 2.0 | 7 | 2.0 | 2 | 2.2 | 4 | 2.3 |
| 18.5-24.9 (healthy) | 216 | 14.6 | 68 | 19.5 | 23 | 24.7 | 39 | 22.5 |
| 25-29.9 (overweight) | 419 | 28.3 | 102 | 29.2 | 31 | 33.3 | 59 | 34.1 |
| 30-39.9 (obese) | 597 | 40.3 | 135 | 38.7 | 27 | 29.0 | 53 | 30.6 |
| ≥ 40 (morbidly obese) | 222 | 15.0 | 37 | 10.6 | 10 | 10.8 | 18 | 10.4 |
| **Immunomodulators** |  |  |  |  |  |  |  |  |
| Yes | 95 | 6.4 | 31 | 8.8 | 5 | 5.4 | 19 | 10.9 |
| No | 1392 | 93.6 | 320 | 91.2 | 88 | 94.6 | 155 | 89.1 |
| **Corticosteroids** |  |  |  |  |  |  |  |  |
| Yes | 991 | 66.6 | 268 | 76.4 | 60 | 64.5 | 131 | 75.3 |
| No | 496 | 33.4 | 83 | 23.6 | 33 | 35.5 | 43 | 24.7 |
| **Remdesivir prior to rand.** |  |  |  |  |  |  |  |  |
| Yes | 878 | 59.0 | 233 | 66.4 | 60 | 64.5 | 112 | 64.4 |
| No | 609 | 41.0 | 118 | 33.6 | 33 | 35.5 | 62 | 35.6 |
| **Pulmonary status** |  |  |  |  |  |  |  |  |
| No O2 | 388 | 26.1 | 62 | 17.7 | 17 | 18.3 | 31 | 17.8 |
| O2 < 4 L/min | 539 | 36.2 | 127 | 36.2 | 25 | 26.9 | 47 | 27.0 |
| O2 ≥ 4 L/min | 415 | 27.9 | 123 | 35.0 | 28 | 30.1 | 59 | 33.9 |
| Non-invasive vent./HFNC | 145 | 9.8 | 39 | 11.1 | 23 | 24.7 | 37 | 21.3 |
| **Borg Dyspnea Scale** |  |  |  |  |  |  |  |  |
| 0-2 (nothing to slight) | 662 | 48.1 | 156 | 47.3 | 31 | 35.6 | 69 | 41.8 |
| 3-4 (mod-somewhat severe) | 443 | 32.2 | 102 | 30.9 | 36 | 41.4 | 53 | 32.1 |
| 5-10 (severe-maximal) | 270 | 19.6 | 72 | 21.8 | 20 | 23.0 | 43 | 26.1 |
| **NEWS** |  |  |  |  |  |  |  |  |
| < 2 | 185 | 12.5 | 26 | 7.4 | 9 | 9.8 | 17 | 9.8 |
| 2-3 | 487 | 32.9 | 109 | 31.1 | 26 | 28.3 | 40 | 23.0 |
| 4-5 | 478 | 32.3 | 118 | 33.6 | 29 | 31.5 | 59 | 33.9 |
| ≥ 6 | 328 | 22.2 | 98 | 27.9 | 28 | 30.4 | 58 | 33.3 |
| **Platelets – med. (IQR) x10^9^/L** | 214 (170 - 273) | | 194 (157 - 248) | | 197 (148 - 244) | | 169 (124 - 230) | |
| **Hemoglobin – med. (IQR) g/dL** | 13.4 (12.2 - 14.5) | | 12.8 (11.5 - 14.1) | | 13.5 (12.1 - 14.4) | | 12.2 (10.6 - 13.8) | |
| **Serum creatinine - med. (IQR) mg/dL** | 0.83 (0.69 - 1.04) | | 0.83 (0.70 - 1.10) | | 0.95 (0.80 - 1.30) | | 1.07 (0.80 - 1.75) | |
| < 1.1 | 1154 | 77.7 | 259 | 74.0 | 57 | 61.3 | 88 | 50.6 |
| 1.1-1.5 | 212 | 14.3 | 54 | 15.4 | 21 | 22.6 | 33 | 19.0 |
| > 1.5 | 120 | 8.1 | 37 | 10.6 | 15 | 16.1 | 53 | 30.5 |
| **eGFR - med. (IQR)** | 94 (73 - 109) | | 89 (68 - 107) | | 79 (51 - 94) | | 68 (38 - 92) | |
| < 60 | 221 | 14.9 | 70 | 20.0 | 29 | 31.2 | 75 | 43.1 |
| ≥ 60 | 1265 | 85.1 | 280 | 80.0 | 64 | 68.8 | 99 | 56.9 |
| **CRP - med. (IQR) mg/dL** | 2.7 (1.3 - 5.1) | | 3.9 (2.0 - 6.2) | | 4.2 (2.1 - 7.2) | | 4.3 (2.4 - 6.9) | |
| < 5 | 1008 | 74.1 | 205 | 63.5 | 47 | 58.0 | 87 | 57.6 |
| 5-7.5 | 177 | 13.0 | 62 | 19.2 | 16 | 19.8 | 33 | 21.9 |
| > 7.5 | 175 | 12.9 | 56 | 17.3 | 18 | 22.2 | 31 | 20.5 |
| **IL-6 - med (IQR) ng/L** | 5.5 (2.2 - 14.0) | | 6.4 (2.7 - 17.3) | | 10.2 (5.9 - 20.4) | | 9.9 (4.1 - 23.6) | |
| ≤ 5.8 | 729 | 51.4 | 158 | 46.3 | 21 | 24.1 | 56 | 35.2 |
| > 5.8 | 690 | 48.6 | 183 | 53.7 | 66 | 75.9 | 103 | 64.8 |
| **D-dimer – med (IQR) mg/L** | 0.89 (0.60 - 1.41) | | 1.09 (0.75 - 1.68) | | 0.98 (0.67 - 1.41) | | 1.20 (0.77 - 2.21) | |
| ≤ 0.93 | 761 | 53.6 | 136 | 39.9 | 38 | 43.7 | 53 | 33.3 |
| > 0.93 | 658 | 46.4 | 205 | 60.1 | 49 | 56.3 | 106 | 66.7 |

IQR=interquartile range, Ag=antigen, Ab=antibody, COPD=chronic obstructive pulmonary disease, BMI=body mass index, HFNC=high flow nasal canula, NEWS=National Early Warning Score, eGFR=estimated glomerular filtration rate, CRP=C-reactive protein, IL-6=interleukin 6 Percentages are calculated among those with available data for each variable

## Figure E2 A/B: Oxygen requirements at D0 by Lymphopenia Trajectory Group and Severe Lymphopenia Trajectory Group

**A: Lymphopenia (ALC < 0.9)**

**
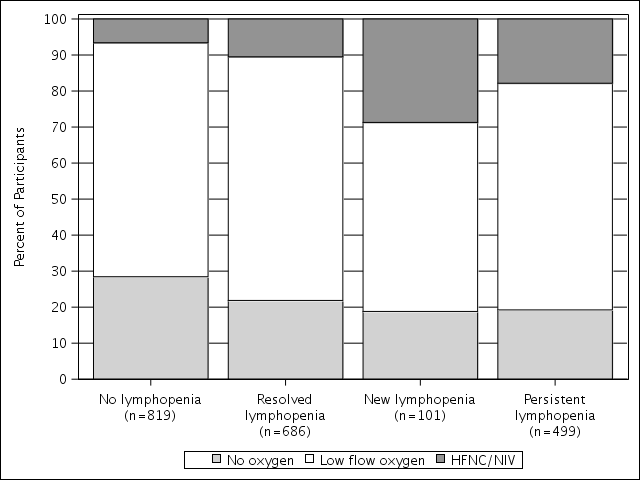
**

HFNC=high flow nasal canula, NIV=noninvasive ventilation

**B: Severe lymphopenia (ALC < 0.56)**

**
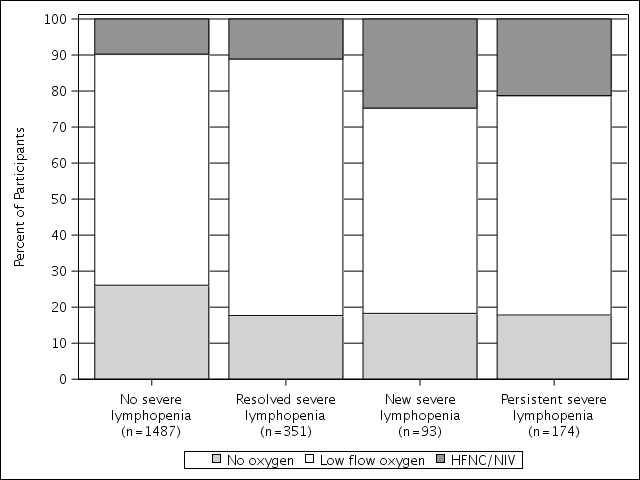
**

HFNC=high flow nasal canula, NIV=noninvasive ventilation

Distribution of baseline oxygen requirements by lymphopenia trajectory group (A) and severe lymphopenia trajectory group (B)

## Figure E3 A/B: Oxygen requirements at D5 by Lymphopenia Trajectory Group and Severe Lymphopenia Trajectory Group

**A: Lymphopenia (ALC < 0.9)**

**
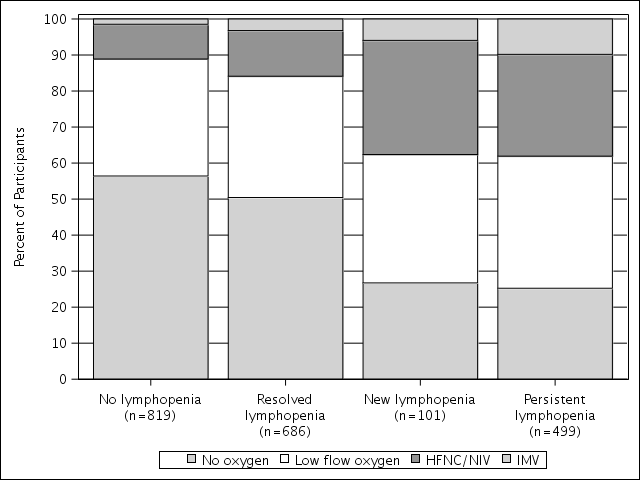
**

HFNC=high flow nasal canula, NIV=noninvasive ventilation, IMV=invasive mechanical ventilation

**B: Severe lymphopenia (ALC < 0.56)**

**
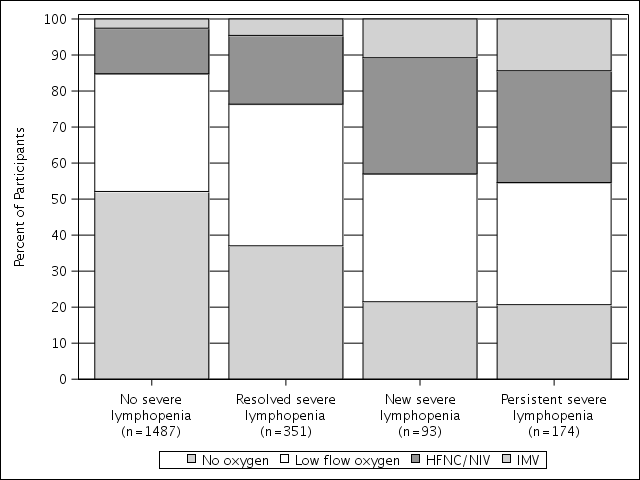
**

HFNC=high flow nasal canula, NIV=noninvasive ventilation, IMV=invasive mechanical ventilation

Distribution of oxygen requirements at Day 5 by lymphopenia trajectory group (A) and severe lymphopenia trajectory group (B)

## Figure E4 A/B: Estimated Glomerular Filtration Rate (eGFR) at D0 by Lymphopenia Trajectory Group and Severe Lymphopenia Trajectory Group

**A: Lymphopenia (ALC < 0.9)**

**
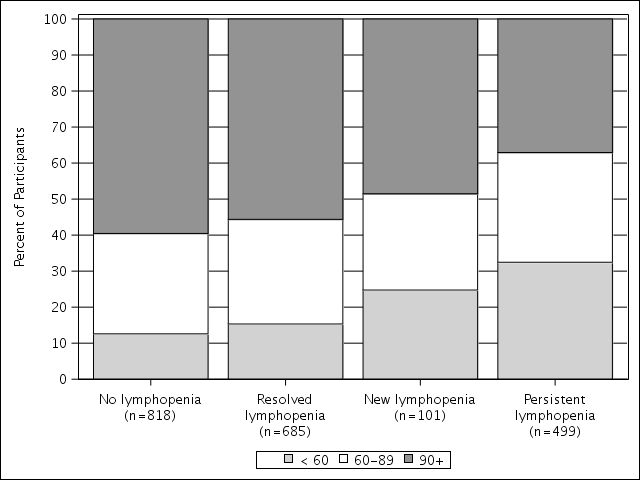
**

**B: Severe lymphopenia (ALC < 0.56)**


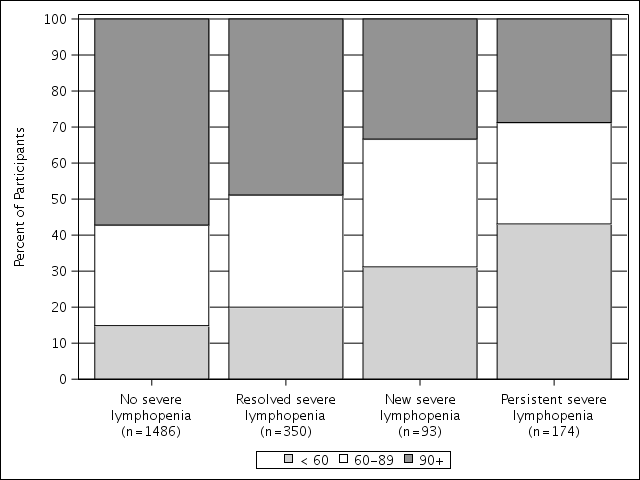


Distribution of Day 0 eGFR by lymphopenia trajectory group (A) and severe lymphopenia trajectory group (B)

## Figure E5 A/B: Estimated Glomerular Filtration Rate (eGFR) at D5 by Lymphopenia Trajectory Group and Severe Lymphopenia Trajectory Group

**A: Lymphopenia (ALC < 0.9)**

**
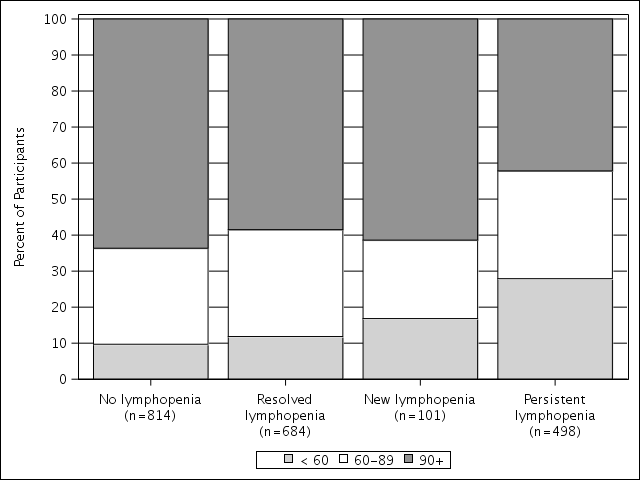
**

**B: Severe lymphopenia (ALC < 0.56)**

**
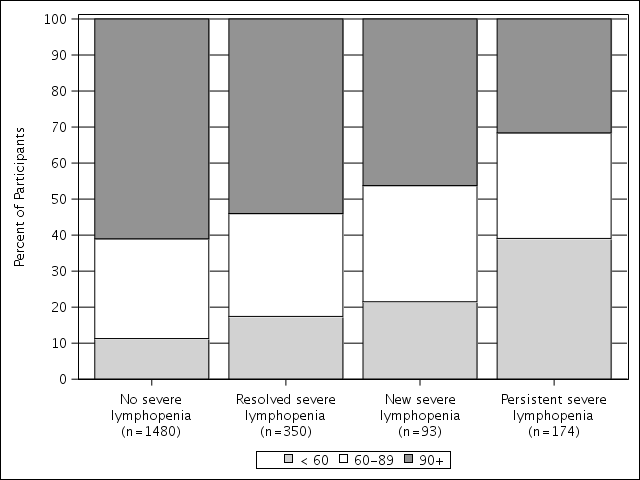
**

Distribution of Day 5 eGFR by lymphopenia trajectory group (A) and severe lymphopenia trajectory group (B)

## Table E5: Sensitivity analysis – Lymphopenia association with outcomes setting those with missing Day 5 Absolute Lymphocyte Count to having lymphopenia

|  | | | | |
| --- | --- | --- | --- | --- |
| **Mortality** | | | | |
|  | | | | |
| **Lymphopenia trajectory groups** | **N Pts.** | **N (%) Deaths** | **HR^*^ (95% CI)** | **p-value** |
| No lymphopenia | 819 | 35 (4.3) | (ref.) |  |
| Resolved lymphopenia | 684 | 39 (5.7) | 1.13 [0.69, 1.87] | 0.62 |
| New lymphopenia | 313 | 20 (6.4) | 1.48 [0.82, 2.68] | 0.19 |
| Persistent lymphopenia | 718 | 136 (18.9) | 2.45 [1.58, 3.79] | <0.001 |
|  |  |  |  |  |
| **Severe lymphopenia trajectory groups** | **N Pts.** | **N (%) Deaths** | **HR^*^ (95% CI)** | **p-value** |
| No severe lymphopenia | 1484 | 89 (6.0) | (ref.) |  |
| Resolved severe lymphopenia | 350 | 35 (10.0) | 1.12 [0.72, 1.74] | 0.61 |
| New severe lymphopenia | 427 | 36 (8.4) | 1.38 [0.88, 2.15] | 0.16 |
| Persistent severe lymphopenia | 273 | 70 (25.6) | 2.65 [1.82, 3.87] | <0.001 |
|  | | | | |
| **Recovery** | | | | |
|  | | | | |
| **Lymphopenia trajectory groups** | **N Pts.** | **N (%) Recovered** | **RRR^†^ (95% CI)** | **p-value** |
| No severe lymphopenia | 819 | 760 (92.8) | (ref.) |  |
| Resolved severe lymphopenia | 684 | 631 (92.3) | 1.05 [0.94, 1.17] | 0.40 |
| New severe lymphopenia | 310 | 276 (89.0) | 1.09 [0.92, 1.29] | 0.30 |
| Persistent severe lymphopenia | 715 | 539 (75.4) | 0.79 [0.70, 0.89] | <0.001 |
|  |  |  |  |  |
| **Severe lymphopenia trajectory groups** | **N Pts.** | **N (%) Recovered** | **RRR^†^ (95% CI)** | **p-value** |
| No severe lymphopenia | 1484 | 1351 (91.0) | (ref.) |  |
| Resolved severe lymphopenia | 350 | 305 (87.1) | 0.88 [0.77, 1.00] | 0.046 |
| New severe lymphopenia | 422 | 372 (88.2) | 1.15 [0.99, 1.32] | 0.06 |
| Persistent severe lymphopenia | 272 | 178 (65.4) | 0.70 [0.58, 0.83] | <0.001 |
| **Secondary Infections** | | | | |
|  |  |  |  |  |
| **Lymphopenia trajectory groups** | **N Pts.** | **N (%) Infections** | **HR^*^ (95% CI)** | **p-value** |
| No severe lymphopenia | 808 | 30 (3.7) | (ref.) |  |
| Resolved severe lymphopenia | 678 | 23 (3.4) | 0.72 [0.40, 1.28] | 0.26 |
| New severe lymphopenia | 303 | 15 (5.0) | 1.10 [0.57, 2.14] | 0.78 |
| Persistent severe lymphopenia | 687 | 86 (12.5) | 1.97 [1.22, 3.18] | 0.006 |
|  |  |  |  |  |
| **Severe lymphopenia trajectory groups** | **N Pts.** | **N (%) Infections** | **HR^*^ (95% CI)** | **p-value** |
| No severe lymphopenia | 1460 | 63 (4.3) | (ref.) |  |
| Resolved severe lymphopenia | 345 | 26 (7.5) | 1.05 [0.64, 1.73] | 0.046 |
| New severe lymphopenia | 412 | 26 (6.3) | 1.17 [0.69, 1.97] | 0.06 |
| Persistent severe lymphopenia | 259 | 39 (15.1) | 2.25 [1.43, 3.54] | <0.001 |
|  | | | | |
| ^*^ Hazard ratio adjusted for age, gender, race/ethnicity, residence, geographical region, date of infection, baseline pulmonary status, Quanterix Ag, SARS-CoV-2 viral load, eGFR, CRP, IL-6, and D-dimer | | | | |
| **^†^** Recovery rate ratio for sustained recovery adjusted for age, gender, race/ethnicity, residence, geographical region, date of infection, baseline pulmonary status, Quanterix Ag, SARS-CoV-2 viral load, eGFR, CRP, IL-6, and D-dimer | | | | |

## Table E6: Sensitivity analysis – Lymphopenia association with outcomes setting those with missing Day 5 Absolute Lymphocyte Count to not having lymphopenia

|  | | | | |
| --- | --- | --- | --- | --- |
| **Mortality** | | | | |
|  | | | | |
| **Lymphopenia trajectory groups** | **N Pts.** | **N (%) Deaths** | **HR^*^ (95% CI)** | **p-value** |
| No lymphopenia | 1031 | 42 (4.1) | (ref.) |  |
| Resolved lymphopenia | 905 | 55 (6.1) | 1.23 [0.79, 1.91] | 0.36 |
| New lymphopenia | 101 | 13 (12.9) | 1.75 [0.90, 3.43] | 0.10 |
| Persistent lymphopenia | 497 | 120 (24.1) | 2.49 [1.65, 3.75] | <0.001 |
|  |  |  |  |  |
| **Severe lymphopenia trajectory groups** | **N Pts.** | **N (%) Deaths** | **HR^*^ (95% CI)** | **p-value** |
| No severe lymphopenia | 1818 | 102 (5.6) | (ref.) |  |
| Resolved severe lymphopenia | 449 | 45 (10.0) | 1.19 [0.80, 1.77] | 0.40 |
| New severe lymphopenia | 93 | 23 (24.7) | 1.67 [0.97, 2.89] | 0.07 |
| Persistent severe lymphopenia | 174 | 60 (34.5) | 2.95 [2.01, 4.32] | <0.001 |
|  | | | | |
| **Recovery** | | | | |
|  | | | | |
| **Lymphopenia trajectory groups** | **N Pts.** | **N (%) Recovered** | **RRR^†^ (95% CI)** | **p-value** |
| No severe lymphopenia | 1028 | 954 (92.8) | (ref.) |  |
| Resolved severe lymphopenia | 902 | 826 (91.6) | 1.06 [0.95, 1.17] | 0.29 |
| New severe lymphopenia | 101 | 82 (81.2) | 0.76 [0.59, 0.97] | 0.026 |
| Persistent severe lymphopenia | 497 | 344 (69.2) | 0.59 [0.52, 0.68] | <0.001 |
|  |  |  |  |  |
| **Severe lymphopenia trajectory groups** | **N Pts.** | **N (%) Recovered** | **RRR^†^ (95% CI)** | **p-value** |
| No severe lymphopenia | 1484 | 1351 (91.0) | (ref.) |  |
| Resolved severe lymphopenia | 350 | 305 (87.1) | 0.88 [0.77, 1.00] | 0.046 |
| New severe lymphopenia | 422 | 372 (88.2) | 1.15 [0.99, 1.32] | 0.06 |
| Persistent severe lymphopenia | 272 | 178 (65.4) | 0.70 [0.58, 0.83] | <0.001 |
| **Secondary Infections** | | | | |
|  |  |  |  |  |
| **Lymphopenia trajectory groups** | **N Pts.** | **N (%) Infections** | **HR^*^ (95% CI)** | **p-value** |
| No severe lymphopenia | 1015 | 34 (3.3) | (ref.) |  |
| Resolved severe lymphopenia | 895 | 31 (3.5) | 0.81 [0.47, 1.38] | 0.44 |
| New severe lymphopenia | 96 | 11 (11.5) | 2.26 [1.10, 4.62] | 0.026 |
| Persistent severe lymphopenia | 470 | 78 (16.6) | 2.80 [1.75, 4.49] | <0.001 |
|  |  |  |  |  |
| **Severe lymphopenia trajectory groups** | **N Pts.** | **N (%) Infections** | **HR^*^ (95% CI)** | **p-value** |
| No severe lymphopenia | 1787 | 71 (4.0) | (ref.) |  |
| Resolved severe lymphopenia | 442 | 30 (6.8) | 1.02 [0.63, 1.64] | 0.95 |
| New severe lymphopenia | 85 | 18 (21.2) | 2.49 [1.36, 4.55] | 0.003 |
| Persistent severe lymphopenia | 162 | 35 (21.6) | 3.55 [2.24, 5.63] | <0.001 |
|  | | | | |
| ^*^ Hazard ratio adjusted for age, gender, race/ethnicity, residence, geographical region, date of infection, baseline pulmonary status, Quanterix Ag, SARS-CoV-2 viral load, eGFR, CRP, IL-6, and D-dimer | | | | |
| **^†^** Recovery rate ratio for sustained recovery adjusted for age, gender, race/ethnicity, residence, geographical region, date of infection, baseline pulmonary status, Quanterix Ag, SARS-CoV-2 viral load, eGFR, CRP, IL-6, and D-dimer | | | | |

## Figure E6: Time to Recovery by lymphopenia trajectory groups (ALC < 0.9)


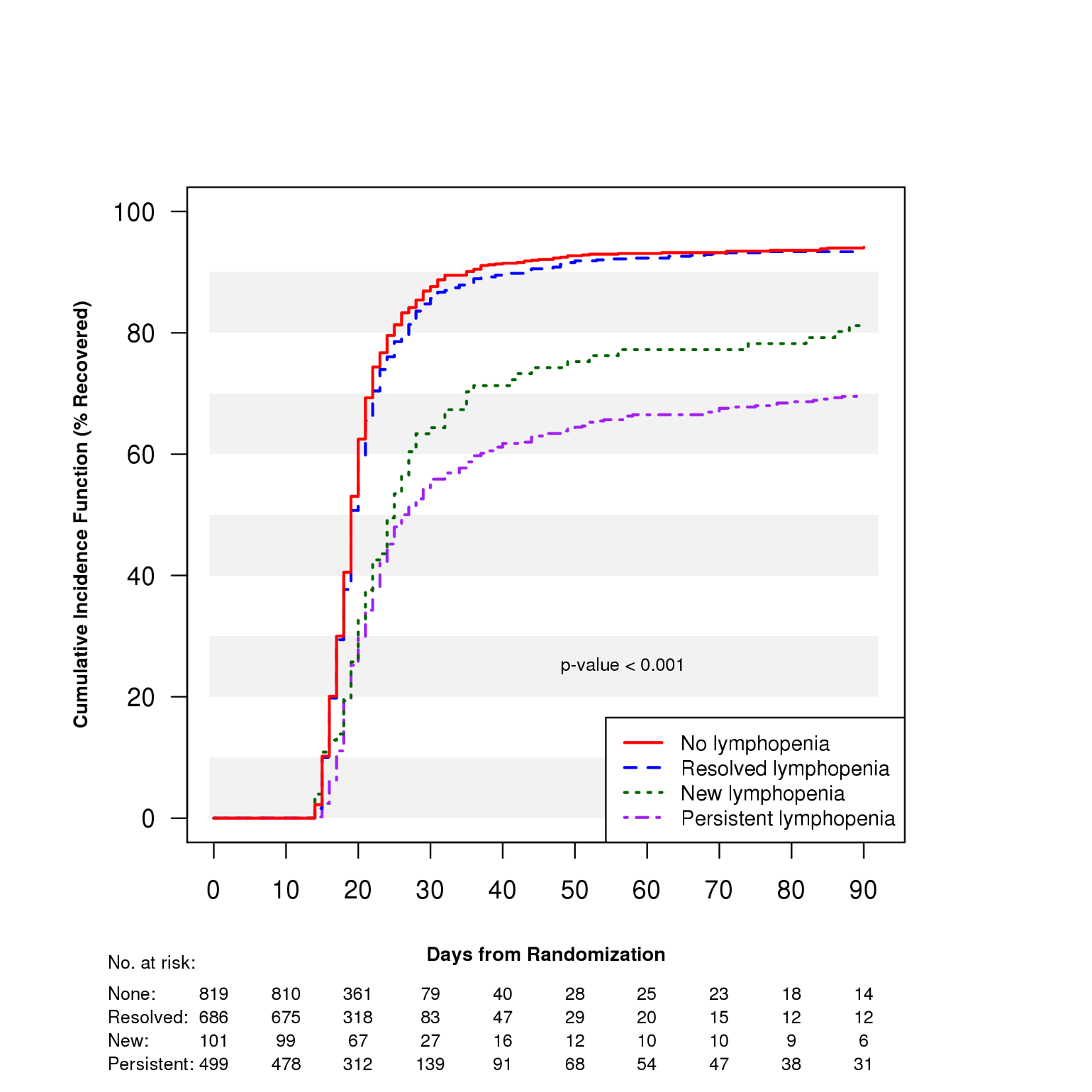


Aalen-Johansen curves for time to recovery by lymphopenia groups.

## Figure E7: Time to Recovery by severe lymphopenia trajectory groups (ALC < 0.56)


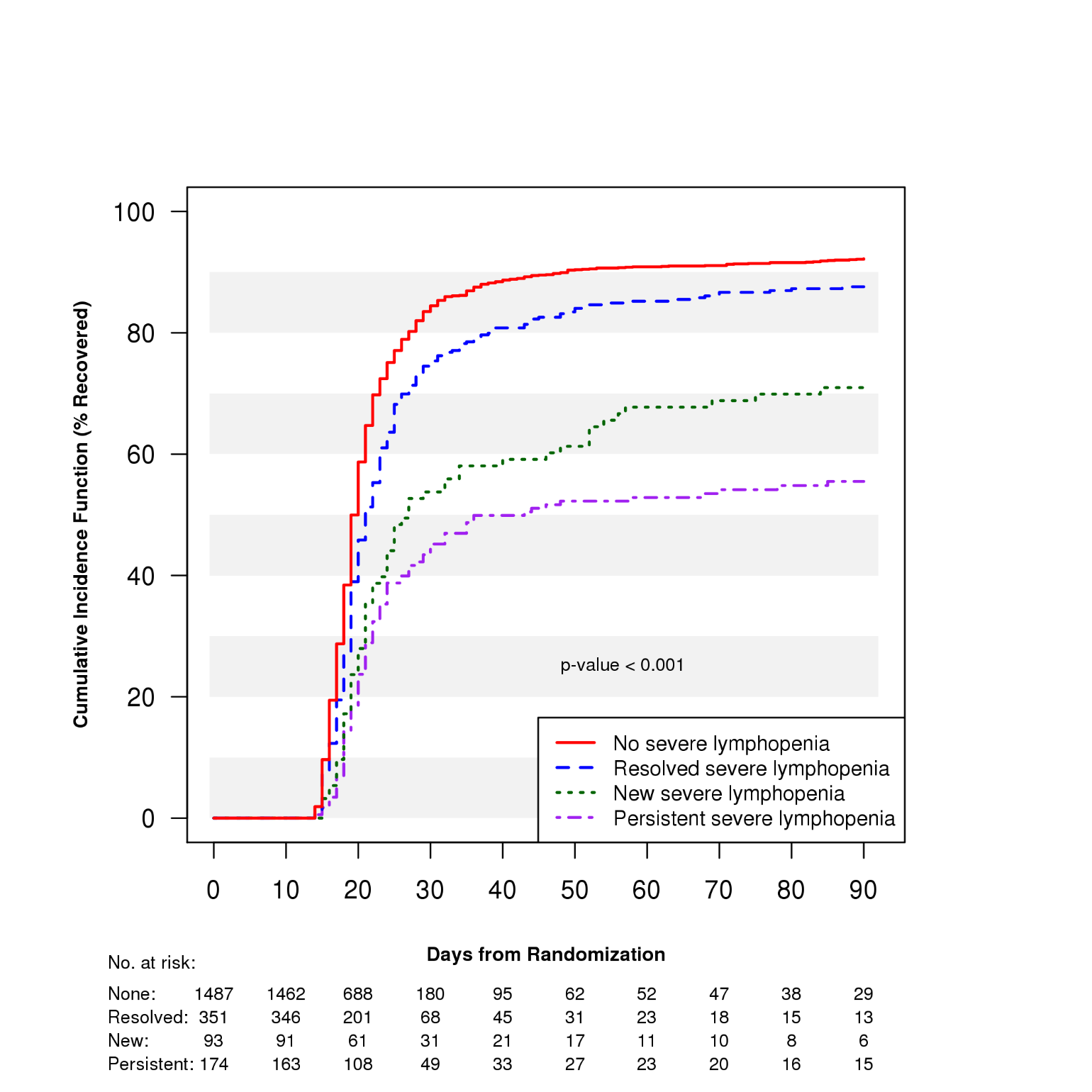


Aalen-Johansen curves for time to recovery by severe lymphopenia groups.

## Table E7: Demographic Factors associated with Day 0 Lymphopenia, New (Day 5) Lymphopenia, or Resolved (Day 5) Lymphopenia

|  | **D0 Lymphopenia** | | **New Lymphopenia compared to No Lymphopenia** | | **Resolved Lymphopenia compared to Persistent Lymphopenia** | |
| --- | --- | --- | --- | --- | --- | --- |
|  | **N Pts.** | **OR (95% CI)^†^** | **N Pts.** | **OR (95% CI)^‡^** | **N Pts.** | **OR (95% CI)^‡^** |
| **Age** |  |  |  |  |  |  |
| 18-39 years | 352 | (ref.) | 151 | (ref.) | 126 | (ref.) |
| 40-49 years | 476 | 1.18 (0.86 - 1.61) | 191 | 2.90 (0.91 - 9.26) | 199 | 0.91 (0.52 - 1.59) |
| 50-59 years | 618 | 1.30 (0.96 - 1.76) | 224 | 4.77 (1.57 - 14.50)****** | 292 | 0.86 (0.51 - 1.46) |
| 60-69 years | 559 | 1.39 (1.01 - 1.92)***** | 191 | 3.84 (1.23 - 11.96)***** | 285 | 0.43 (0.26 - 0.73)***** |
| 70-79 years | 399 | 1.38 (0.97 - 1.96) | 117 | 5.33 (1.60 - 17.72)****** | 199 | 0.52 (0.29 - 0.91)***** |
| ≥ 80 years | 175 | 1.64 (1.02 - 2.61)***** | 46 | 3.46 (0.71 - 16.93) | 84 | 0.56 (0.28 - 1.15) |
| **Race/Ethnicity** |  |  |  |  |  |  |
| White | 1280 | (ref.) | 425 | (ref.) | 618 | (ref.) |
| Asian | 119 | 1.20 (0.71 - 2.03) | 42 | 2.56 (0.74 - 8.90) | 64 | 1.13 (0.51 - 2.50) |
| Black | 619 | 0.84 (0.65 - 1.09) | 234 | 0.44 (0.19 - 1.06) | 264 | 1.26 (0.86 - 1.83) |
| Hispanic | 474 | 0.70 (0.54 - 0.90)****** | 184 | 1.33 (0.65 - 2.70) | 204 | 1.18 (0.79 - 1.79) |
| Other | 87 | 0.65 (0.40 - 1.06) | 35 | 0.92 (0.25 - 3.44) | 35 | 1.29 (0.57 - 2.93) |
| **Sex** |  |  |  |  |  |  |
| Male | 1484 | (ref.) | 484 | (ref.) | 728 | (ref.) |
| Female | 1095 | 0.67 (0.56 - 0.80)******* | 436 | 0.71 (0.42 - 1.19) | 457 | 1.42 (1.07 - 1.89) |
| **Residence** |  |  |  |  |  |  |
| Independent w/o assistance | 2431 | 0.73 (0.49 - 1.09) | 877 | 1.73 (0.36 - 8.22) | 1109 | 0.63 (0.36 - 1.11) |
| All other | 148 | (ref.) | 43 | (ref.) | 76 | (ref.) |
| **Region** |  |  |  |  |  |  |
| United States | 2019 | (ref.) | 638 | (ref.) | 950 | (ref.) |
| Europe | 389 | 0.68 (0.52 - 0.88)****** | 187 | 0.44 (0.19 - 1.00) | 172 | 1.23 (0.81 - 1.86) |
| Africa | 129 | 0.39 (0.24 - 0.62)******* | 81 | 1.62 (0.43 - 6.07) | 37 | 0.95 (0.40 - 2.22) |
| Asia | 42 | 0.76 (0.33 - 1.77) | 14 | 1.13 (0.18 - 7.19) | 26 | 0.90 (0.28 - 2.87) |
| **Date of Infection** |  |  |  |  |  |  |
| Pre 2021 | 401 | 1.49 (1.09 - 2.04)***** | 124 | 0.61 (0.23 - 1.60) | 197 | 0.82 (0.52 - 1.30) |
| Jan-Jun 2021 | 1034 | 1.08 (0.88 - 1.33) | 364 | 0.87 (0.48 - 1.59) | 472 | 1.16 (0.83 - 1.62) |
| Jul-Dec 2021 | 1144 | (ref.) | 432 | (ref.) | 516 | (ref.) |
|  | | | | | | |
| * Significant p-values are denoted as follows next to parentheses: * p-value < 0.05, ** p-value < 0.01, *** p-value < 0.001  ^†^ Adjusted for age, sex, race/ethnicity, residence, geographical region, date of infection, baseline pulmonary status, Quanterix Ag, SARS-CoV-2 viral load, eGFR, CRP, IL-6, D-dimer, corticosteroid use, immunomodulator use, and remdesivir use | | | | | | |
| ^‡^ Adjusted for age, sex, race/ethnicity, residence, geographical region, date of infection, baseline pulmonary status, Quanterix Ag, SARS-CoV-2 viral load, eGFR, CRP, IL-6, D-dimer, D0 and D5 corticosteroid use, D0 and D5 immunomodulator use, D0 and D5 remdesivir use, and randomized treatment group | | | | | | |

## Table E8: COVID-19 Characteristics associated with Day 0 Lymphopenia, New (Day 5) Lymphopenia, or Resolved (Day 5) Lymphopenia

|  | **D0 Lymphopenia** | | **New Lymphopenia compared to No Lymphopenia** | | **Resolved Lymphopenia compared to Persistent Lymphopenia** | |
| --- | --- | --- | --- | --- | --- | --- |
|  | **N Pts.** | **OR (95% CI)^†^** | **N Pts.** | **OR (95% CI)^‡^** | **N Pts.** | **OR (95% CI)^‡^** |
| **Symptom duration (days)** |  |  |  |  |  |  |
| < 5 | 393 | (ref.) | 142 | (ref.) | 172 | (ref.) |
| 5 - 7 | 749 | 1.11 (0.83 - 1.48) | 253 | 0.41 (0.19 - 0.90)***** | 348 | 1.01 (0.65 - 1.59) |
| 8 - 10 | 1070 | 1.33 (1.01 - 1.76)***** | 392 | 0.45 (0.22 - 0.94)***** | 487 | 0.91 (0.58 - 1.41) |
| > 10 | 367 | 1.39 (0.99 - 1.97) | 133 | 0.93 (0.38 - 2.30) | 178 | 1.01 (0.59 - 1.73) |
| **Number of vaccine doses** |  |  |  |  |  |  |
| 0 | 2114 | (ref.) | 762 | (ref.) | 965 | (ref.) |
| 1 | 179 | 1.22 (0.85 - 1.76) | 58 | 0.87 (0.26 - 2.90) | 87 | 0.65 (0.38 - 1.10) |
| 2 | 264 | 0.92 (0.66 - 1.27) | 91 | 0.85 (0.36 - 2.05) | 124 | 0.68 (0.42 - 1.12) |
| **SARS-CoV-2 viral load** |  |  |  |  |  |  |
| Negative | 329 | (ref.) | 133 | (ref.) | 131 | (ref.) |
| < 35,000 copies/mL | 1070 | 1.04 (0.78 - 1.39) | 405 | 1.95 (0.70 - 5.47) | 480 | 0.85 (0.52 - 1.38) |
| 35,000+ copies/mL | 1073 | 1.13 (0.84 - 1.54) | 349 | 1.24 (0.42 - 3.62) | 542 | 0.67 (0.40 - 1.10) |
| **Anti-spike Ab status** |  |  |  |  |  |  |
| Positive | 1289 | 0.93 (0.75 - 1.14) | 490 | 1.19 (0.65 - 2.19) | 558 | 1.27 (0.93 - 1.74) |
| Negative | 1213 | (ref.) | 409 | (ref.) | 600 | (ref.) |
| **Anti-N Ab status** |  |  |  |  |  |  |
| Positive | 1559 | 1.09 (0.89 - 1.35) | 577 | 0.93 (0.51 - 1.68) | 706 | 1.14 (0.84 - 1.54) |
| Negative | 944 | (ref.) | 323 | (ref.) | 452 | (ref.) |
| **Quanterix Ag (ng/L)** |  |  |  |  |  |  |
| < 200 | 598 | (ref.) | 272 | (ref.) | 198 | (ref.) |
| 200 - 1499 | 670 | 1.52 (1.18 - 1.95)****** | 269 | 1.42 (0.62 - 3.21) | 284 | 0.94 (0.59 - 1.49) |
| 1500 - 4499 | 583 | 2.27 (1.73 - 2.98)******* | 178 | 2.52 (1.10 - 5.74)***** | 290 | 0.83 (0.53 - 1.32) |
| ≥ 4500 | 651 | 2.66 (1.99 - 3.55)******* | 180 | 2.03 (0.87 - 4.75) | 386 | 0.62 (0.39 - 0.99)***** |
|  |  |  |  |  |  |  |
| 1000+ | 1434 | 2.02 (1.65 - 2.46)******* | 427 | 2.02 (1.12 - 3.64)***** | 764 | 0.83 (0.61 - 1.14) |
| < 1000 | 1068 | (ref.) | 472 | (ref.) | 394 | (ref.) |
|  |  |  |  |  |  |  |
| * Significant p-values are denoted as follows next to parentheses: * p-value < 0.05, ** p-value < 0.01, *** p-value < 0.001  ^†^ Adjusted for age, sex, race/ethnicity, residence, geographical region, date of infection, baseline pulmonary status, Quanterix Ag, SARS-CoV-2 viral load, eGFR, CRP, IL-6, D-dimer, corticosteroid use, immunomodulator use, and remdesivir use | | | | | | |
| ^‡^ Adjusted for age, sex, race/ethnicity, residence, geographical region, date of infection, baseline pulmonary status, Quanterix Ag, SARS-CoV-2 viral load, eGFR, CRP, IL-6, D-dimer, D0 and D5 corticosteroid use, D0 and D5 immunomodulator use, D0 and D5 remdesivir use, and randomized treatment group  * p-value < 0.05, ** p-value < 0.01, *** p-value < 0.001  Ab=antibody, Ag=antigen | | | | | | |

## Table E9: Comorbid Conditions associated with Day 0 Lymphopenia, New (Day 5) Lymphopenia, or Resolved (Day 5) Lymphopenia

|  | **D0 Lymphopenia** | | **New Lymphopenia compared to No Lymphopenia** | | **Resolved Lymphopenia compared to Persistent Lymphopenia** | |
| --- | --- | --- | --- | --- | --- | --- |
|  | **N Pts.** | **OR (95% CI)^†^** | **N Pts.** | **OR (95% CI)^‡^** | **N Pts.** | **OR (95% CI)^‡^** |
| **BMI** |  |  |  |  |  |  |
| < 18.5 (underweight) | 47 | 0.70 (0.35 - 1.40) | 24 | 0.44 (0.05 - 3.92) | 18 | 2.77 (0.87 - 8.84) |
| 18.5-24.9 (healthy) | 422 | (ref.) | 133 | (ref.) | 213 | (ref.) |
| 25-29.9 (overweight) | 743 | 0.71 (0.53 - 0.94)***** | 254 | 1.08 (0.47 - 2.53) | 357 | 1.59 (1.05 - 2.39)***** |
| 30-39.9 (obese) | 985 | 0.57 (0.43 - 0.75)******* | 363 | 1.08 (0.48 - 2.47) | 449 | 2.09 (1.38 - 3.16)******* |
| ≥ 40 (morbidly obese) | 374 | 0.54 (0.38 - 0.77)******* | 143 | 1.01 (0.35 - 2.93) | 144 | 1.90 (1.11 - 3.25)***** |
| **Asthma** |  |  |  |  |  |  |
| Yes | 255 | 1.04 (0.77 - 1.41) | 92 | 0.66 (0.21 - 2.03) | 104 | 0.79 (0.50 - 1.26) |
| No | 2324 | (ref.) | 828 | (ref.) | 1081 | (ref.) |
| **COPD** |  |  |  |  |  |  |
| Yes | 161 | 0.79 (0.55 - 1.14) | 56 | 1.03 (0.32 - 3.25) | 70 | 1.14 (0.66 - 1.99) |
| No | 2418 | (ref.) | 864 | (ref.) | 1115 | (ref.) |
| **Diabetes** |  |  |  |  |  |  |
| Yes | 726 | 0.97 (0.79 - 1.19) | 232 | 1.39 (0.80 - 2.41) | 349 | 1.10 (0.80 - 1.50) |
| No | 1853 | (ref.) | 688 | (ref.) | 836 | (ref.) |
| **Heart failure** |  |  |  |  |  |  |
| Yes | 114 | 1.29 (0.81 - 2.08) | 27 | 5.16 (1.58 - 16.77)****** | 62 | 1.09 (0.60 - 1.97) |
| No | 2465 | (ref.) | 893 | (ref.) | 1123 | (ref.) |
| **Hypertension** |  |  |  |  |  |  |
| Yes | 1178 | 1.09 (0.89 - 1.32) | 368 | 1.45 (0.82 - 2.55) | 582 | 1.12 (0.83 - 1.50) |
| No | 1401 | (ref.) | 552 | (ref.) | 603 | (ref.) |
| **Renal impairment** |  |  |  |  |  |  |
| Yes | 257 | 1.75 (1.20 - 2.56)****** | 57 | 1.49 (0.58 - 3.86) | 155 | 0.69 (0.43 - 1.11) |
| No | 2322 | (ref.) | 863 | (ref.) | 1030 | (ref.) |
| **HIV** |  |  |  |  |  |  |
| Yes | 41 | 0.54 (0.26 - 1.11) | 21 | 0.94 (0.11 - 8.00) | 14 | 1.44 (0.43 - 4.85) |
| No | 2538 | (ref.) | 899 | (ref.) | 1171 | (ref.) |
| **Other immune suppression** |  |  |  |  |  |  |
| Yes | 80 | 1.57 (0.91 - 2.71) | 20 | 0.51 (0.08 - 3.12) | 50 | 0.49 (0.25 - 0.98)***** |
| No | 2499 | (ref.) | 900 | (ref.) | 1135 | (ref.) |
| **Malignancy** |  |  |  |  |  |  |
| Yes | 105 | 2.05 (1.23 - 3.39)****** | 23 | 0.19 (0.02 - 1.71) | 64 | 0.31 (0.16 - 0.59)******* |
| No | 2474 | (ref.) | 897 | (ref.) | 1121 | (ref.) |
| **Number of comorbidities** |  |  |  |  |  |  |
| 0 | 936 | (ref.) | 391 | (ref.) | 386 | (ref.) |
| 1 | 779 | 1.12 (0.89 - 1.41) | 267 | 1.10 (0.55 - 2.18) | 366 | 0.87 (0.60 - 1.26) |
| 2+ | 560 | 1.12 (0.86 - 1.45) | 186 | 1.49 (0.72 - 3.11) | 264 | 0.94 (0.63 - 1.42) |
| * Significant p-values are denoted as follows next to parentheses: * p-value < 0.05, ** p-value < 0.01, *** p-value < 0.001  ^†^ Adjusted for age, sex, race/ethnicity, residence, geographical region, date of infection, baseline pulmonary status, Quanterix Ag, SARS-CoV-2 viral load, eGFR, CRP, IL-6, D-dimer, corticosteroid use, immunomodulator use, and remdesivir use | | | | | | |
| ^‡^ Adjusted for age, sex, race/ethnicity, residence, geographical region, date of infection, baseline pulmonary status, Quanterix Ag, SARS-CoV-2 viral load, eGFR, CRP, IL-6, D-dimer, D0 and D5 corticosteroid use, D0 and D5 immunomodulator use, D0 and D5 remdesivir use, and randomized treatment group  BMI=body mass index | | | | | | |

## Table E10: Concomitant Medications associated with Day 0 Lymphopenia, New (Day 5) Lymphopenia, or Resolved (Day 5) Lymphopenia

|  | **D0 Lymphopenia** | | **New Lymphopenia compared to No Lymphopenia** | | **Resolved Lymphopenia compared to Persistent Lymphopenia** | |
| --- | --- | --- | --- | --- | --- | --- |
|  | **N Pts.** | **OR (95% CI)^†^** | **N Pts.** | **OR (95% CI)^‡^** | **N Pts.** | **OR (95% CI)^‡^** |
| **Remdesivir use prior to randomization** |  |  |  |  |  |  |
| Yes | 1574 | 0.92 (0.75 - 1.13) | 535 | 1.14 (0.62 - 2.11) | 748 | 0.74 (0.53 - 1.03) |
| No | 1005 | (ref.) | 385 | (ref.) | 437 | (ref.) |
| **Corticosteroids** |  |  |  |  |  |  |
| Yes | 1756 | 1.31 (1.05 - 1.62)***** | 581 | 1.27 (0.65 - 2.49) | 869 | 1.33 (0.93 - 1.89) |
| No | 823 | (ref.) | 339 | (ref.) | 316 | (ref.) |
| **Antibiotics** |  |  |  |  |  |  |
| Yes | 810 | 1.07 (0.87 - 1.32) | 295 | 1.03 (0.56 - 1.89) | 392 | 0.84 (0.62 - 1.13) |
| No | 1769 | (ref.) | 625 | (ref.) | 793 | (ref.) |
| **ACE inhibitors** |  |  |  |  |  |  |
| Yes | 268 | 0.95 (0.71 - 1.28) | 81 | 0.95 (0.37 - 2.45) | 129 | 1.13 (0.73 - 1.75) |
| No | 2311 | (ref.) | 839 | (ref.) | 1056 | (ref.) |
| **ARBs** |  |  |  |  |  |  |
| Yes | 205 | 1.23 (0.87 - 1.73) | 59 | 1.79 (0.73 - 4.40) | 106 | 0.87 (0.54 - 1.40) |
| No | 2374 | (ref.) | 861 | (ref.) | 1079 | (ref.) |
| **Aspirin** |  |  |  |  |  |  |
| Yes | 385 | 1.38 (1.06 - 1.81)***** | 101 | 2.57 (1.26 - 5.26)****** | 210 | 1.05 (0.73 - 1.51) |
| No | 2194 | (ref.) | 819 | (ref.) | 975 | (ref.) |
| **Other antiplatelets** |  |  |  |  |  |  |
| Yes | 155 | 1.20 (0.80 - 1.80) | 47 | 0.92 (0.31 - 2.71) | 71 | 1.31 (0.73 - 2.34) |
| No | 2424 | (ref.) | 873 | (ref.) | 1114 | (ref.) |
| **Heparin** |  |  |  |  |  |  |
| None | 815 | (ref.) | 288 | (ref.) | 348 | (ref.) |
| Prophylactic dose | 1515 | 1.06 (0.86 - 1.32) | 526 | 1.25 (0.66 - 2.36) | 730 | 1.07 (0.77 - 1.47) |
| Intermediate/therapeutic dose | 249 | 0.77 (0.55 - 1.08) | 106 | 0.70 (0.25 - 1.98) | 107 | 1.53 (0.89 - 2.64) |
| **DOAC** |  |  |  |  |  |  |
| Yes | 112 | 1.16 (0.74 - 1.83) | 33 | 0.79 (0.16 - 3.81) | 57 | 0.75 (0.39 - 1.43) |
| No | 2467 | (ref.) | 887 | (ref.) | 1128 | (ref.) |
| **Antirejection** |  |  |  |  |  |  |
| Yes | 101 | 2.69 (1.53 - 4.73)******* | 17 | 5.50 (1.54 - 19.60)****** | 66 | 0.27 (0.14 - 0.54)******* |
| No | 2478 | (ref.) | 903 | (ref.) | 1119 | (ref.) |
| **Immunomodulators** |  |  |  |  |  |  |
| Yes | 168 | 1.26 (0.86 - 1.86) | 53 | 0.47 (0.14 - 1.55) | 97 | 1.40 (0.80 - 2.44) |
| No | 2411 | (ref.) | 867 | (ref.) | 1088 | (ref.) |
| **NSAIDs** |  |  |  |  |  |  |
| Yes | 188 | 1.04 (0.73 - 1.48) | 64 | 0.32 (0.07 - 1.45) | 85 | 1.23 (0.69 - 2.19) |
| No | 2391 | (ref.) | 856 | (ref.) | 1100 | (ref.) |
| * Significant p-values are denoted as follows next to parentheses: * p-value < 0.05, ** p-value < 0.01, *** p-value < 0.001  ^†^ Adjusted for age, sex, race/ethnicity, residence, geographical region, date of infection, baseline pulmonary status, Quanterix Ag, SARS-CoV-2 viral load, eGFR, CRP, IL-6, D-dimer, corticosteroid use, immunomodulator use, and remdesivir use | | | | | | |
| ^‡^ Adjusted for age, sex, race/ethnicity, residence, geographical region, date of infection, baseline pulmonary status, Quanterix Ag, SARS-CoV-2 viral load, eGFR, CRP, IL-6, D-dimer, D0 and D5 corticosteroid use, D0 and D5 immunomodulator use, D0 and D5 remdesivir use, and randomized treatment group  ACE=angiotensin-converting enzyme, ARB=angiotensin receptor blockers, DOAC=direct oral anticoagulant, NSAID=non-steroidal anti-inflammatory drug | | | | | | |

## Table E11: COVID-19 Severity Factors associated with Day 0 Lymphopenia, New (Day 5) Lymphopenia, or Resolved (Day 5) Lymphopenia

|  | **D0 Lymphopenia** | | **New Lymphopenia compared to No Lymphopenia** | | **Resolved Lymphopenia compared to Persistent Lymphopenia** | |
| --- | --- | --- | --- | --- | --- | --- |
|  | **N Pts.** | **OR (95% CI)^†^** | **N Pts.** | **OR (95% CI)^‡^** | **N Pts.** | **OR (95% CI)^‡^** |
| **Pulmonary status** |  |  |  |  |  |  |
| No O2 | 644 | (ref.) | 252 | (ref.) | 246 | (ref.) |
| O2 < 4 L/min | 932 | 1.04 (0.81 - 1.34) | 332 | 0.84 (0.37 - 1.92) | 406 | 1.31 (0.87 - 1.97) |
| O2 ≥ 4 L/min | 719 | 1.28 (0.97 - 1.68) | 253 | 1.38 (0.60 - 3.17) | 372 | 0.93 (0.61 - 1.43) |
| NIV/HFNC | 284 | 1.47 (1.00 - 2.17)***** | 83 | 5.04 (1.90 - 13.35)****** | 161 | 0.71 (0.40 - 1.26) |
| **Borg Dyspnea Scale** |  |  |  |  |  |  |
| 0-2 (nothing to slight) | 1133 | (ref.) | 416 | (ref.) | 502 | (ref.) |
| 3-4 (mod-somewhat severe) | 773 | 0.91 (0.73 - 1.13) | 280 | 0.77 (0.41 - 1.45) | 354 | 1.06 (0.75 - 1.48) |
| 5-10 (severe-maximal) | 467 | 1.10 (0.84 - 1.43) | 152 | 0.69 (0.32 - 1.52) | 253 | 1.05 (0.71 - 1.54) |
| **NEWS** |  |  |  |  |  |  |
| < 2 | 311 | (ref.) | 125 | (ref.) | 112 | (ref.) |
| 2-3 | 838 | 1.07 (0.74 - 1.53) | 305 | 0.52 (0.16 - 1.68) | 357 | 0.68 (0.36 - 1.25) |
| 4-5 | 821 | 1.08 (0.74 - 1.58) | 291 | 0.49 (0.15 - 1.64) | 393 | 0.70 (0.37 - 1.34) |
| ≥ 6 | 597 | 1.24 (0.82 - 1.88) | 196 | 0.65 (0.19 - 2.24) | 316 | 0.68 (0.34 - 1.36) |
| **Serum creatinine mg/dL** |  |  |  |  |  |  |
| < 1.1 | 1902 | (ref.) | 726 | (ref.) | 832 | (ref.) |
| 1.1-1.5 | 400 | 1.22 (0.94 - 1.59) | 118 | 1.30 (0.57 - 2.92) | 202 | 0.67 (0.46 - 0.98)***** |
| > 1.5 | 274 | 1.32 (0.95 - 1.84) | 75 | 4.07 (1.77 - 9.32)******* | 150 | 0.30 (0.19 - 0.49)******* |
| **eGFR** |  |  |  |  |  |  |
| < 60 | 497 | 1.31 (1.01 - 1.69)***** | 128 | 2.72 (1.36 - 5.42)****** | 267 | 0.48 (0.34 - 0.69)******* |
| ≥ 60 | 2079 | (ref.) | 791 | (ref.) | 917 | (ref.) |
| **CRP (mg/L)** |  |  |  |  |  |  |
| < 5 | 1633 | (ref.) | 650 | (ref.) | 697 | (ref.) |
| 5-7.5 | 351 | 1.48 (1.14 - 1.93)****** | 97 | 0.49 (0.19 - 1.24) | 191 | 0.88 (0.61 - 1.26) |
| > 7.5 | 330 | 1.32 (1.01 - 1.73)***** | 98 | 1.01 (0.51 - 2.02) | 182 | 1.02 (0.69 - 1.49) |
| **IL-6 (ng/L)** |  |  |  |  |  |  |
| ≤ 5.8 | 1207 | (ref.) | 441 | (ref.) | 523 | (ref.) |
| > 5.8 | 1227 | 0.66 (0.54 - 0.81)******* | 438 | 2.33 (1.28 - 4.23)****** | 604 | 0.67 (0.49 - 0.91)****** |
| **D-dimer (mg/L)** |  |  |  |  |  |  |
| ≤ 0.93 | 1213 | (ref.) | 484 | (ref.) | 504 | (ref.) |
| > 0.93 | 1221 | 1.42 (1.18 - 1.72)******* | 395 | 1.11 (0.64 - 1.95) | 623 | 0.81 (0.61 - 1.08) |
| * Significant p-values are denoted as follows next to parentheses: * p-value < 0.05, ** p-value < 0.01, *** p-value < 0.001  ^†^ Adjusted for age, sex, race/ethnicity, residence, geographical region, date of infection, baseline pulmonary status, Quanterix Ag, SARS-CoV-2 viral load, eGFR, CRP, IL-6, D-dimer, corticosteroid use, immunomodulator use, and remdesivir use | | | | | | |
| ^‡^ Adjusted for age, sex, race/ethnicity, residence, geographical region, date of infection, baseline pulmonary status, Quanterix Ag, SARS-CoV-2 viral load, eGFR, CRP, IL-6, D-dimer, D0 and D5 corticosteroid use, D0 and D5 immunomodulator use, D0 and D5 remdesivir use, and randomized treatment group  HFNC=high flow nasal canula, NEWS=National Early Warning Score, eGFR=estimated glomerular filtration rate, CRP=C-reactive protein, IL-6=interleukin 6 | | | | | | |

## Table E12: Demographic, clinical, and COVID-19 related factors associated with Day 0 Severe Lymphopenia, New (Day 5) Severe Lymphopenia, or Resolved (Day 5) Severe Lymphopenia

|  | **D0 Lymphopenia** | | **New Severe Lymphopenia compared to No Severe Lymphopenia** | | **Resolved Severe Lymphopenia compared to Persistent Severe Lymphopenia** | |
| --- | --- | --- | --- | --- | --- | --- |
|  | **N Pts.** | **OR (95% CI)^†^** | **N Pts.** | **OR (95% CI)^‡^** | **N Pts.** | **OR (95% CI)^‡^** |
| **Age** |  |  |  |  |  |  |
| 18-39 years | 352 | (ref.) | 235 | (ref.) | 42 | (ref.) |
| 40-49 years | 476 | 1.28 (0.86 - 1.93) | 319 | 1.15 (0.34 - 3.90) | 71 | 0.71 (0.24 - 2.12) |
| 50-59 years | 618 | 1.49 (1.01 - 2.19)***** | 388 | 2.26 (0.76 - 6.77) | 128 | 0.72 (0.26 - 1.99) |
| 60-69 years | 559 | 1.79 (1.21 - 2.65)****** | 328 | 3.59 (1.23 - 10.53)***** | 148 | 0.36 (0.13 - 0.98)***** |
| 70-79 years | 399 | 1.53 (1.00 - 2.33)***** | 220 | 3.45 (1.11 - 10.72)***** | 96 | 0.40 (0.14 - 1.14) |
| ≥ 80 years | 175 | 1.63 (0.97 - 2.74) | 90 | 2.62 (0.62 - 11.11) | 40 | 0.18 (0.05 - 0.61)****** |
| **Race/Ethnicity** |  |  |  |  |  |  |
| White | 1280 | (ref.) | 758 | (ref.) | 285 | (ref.) |
| Asian | 119 | 0.88 (0.49 - 1.59) | 84 | 1.22 (0.37 - 4.10) | 22 | 1.39 (0.33 - 5.79) |
| Black | 619 | 0.87 (0.66 - 1.15) | 375 | 0.14 (0.05 - 0.41)******* | 123 | 1.03 (0.57 - 1.86) |
| Hispanic | 474 | 0.66 (0.49 - 0.90)****** | 306 | 0.62 (0.30 - 1.31) | 82 | 0.76 (0.39 - 1.50) |
| Other | 87 | 0.74 (0.41 - 1.34) | 57 | 1.10 (0.35 - 3.49) | 13 | 0.76 (0.17 - 3.33) |
| **Sex** |  |  |  |  |  |  |
| Male | 1484 | (ref.) | 893 | (ref.) | 319 | (ref.) |
| Female | 1095 | 0.81 (0.66 - 0.99)***** | 687 | 0.54 (0.32 - 0.92)***** | 206 | 1.25 (0.78 - 1.99) |
| **Region** |  |  |  |  |  |  |
| United States | 2019 | (ref.) | 1140 | (ref.) | 448 | (ref.) |
| Europe | 389 | 0.53 (0.38 - 0.74)******* | 302 | 0.50 (0.24 - 1.06) | 57 | 1.21 (0.56 - 2.62) |
| Africa | 129 | 0.43 (0.23 - 0.79)****** | 105 | 1.61 (0.25 - 10.11) | 13 | 0.83 (0.18 - 3.90) |
| Asia | 42 | 0.50 (0.18 - 1.39) | 33 | 0.81 (0.15 - 4.37) | 7 | 0.21 (0.02 - 1.86) |
| **Anti-spike Ab status** |  |  |  |  |  |  |
| Positive | 1289 | 0.78 (0.62 - 0.98)***** | 820 | 0.86 (0.49 - 1.52) | 228 | 1.92 (1.11 - 3.34)***** |
| Negative | 1213 | (ref.) | 725 | (ref.) | 284 | (ref.) |
| **Quanterix Ag (ng/L)** |  |  |  |  |  |  |
| < 200 | 598 | (ref.) | 389 | (ref.) | 81 | (ref.) |
| 200 - 1499 | 670 | 1.08 (0.79 - 1.48) | 451 | 0.79 (0.31 - 1.99) | 102 | 0.58 (0.26 - 1.32) |
| 1500 - 4499 | 583 | 1.55 (1.13 - 2.12)****** | 338 | 2.62 (1.15 - 5.93)***** | 130 | 0.91 (0.41 - 2.03) |
| ≥ 4500 | 651 | 1.66 (1.19 - 2.30)** | 367 | 1.86 (0.79 - 4.41) | 199 | 0.48 (0.22 - 1.05) |
|  |  |  |  |  |  |  |
| 1000+ | 1434 | 1.55 (1.23 - 1.95)******* | 832 | 2.33 (1.26 - 4.30)****** | 359 | 0.80 (0.47 - 1.37) |
| < 1000 | 1068 | (ref.) | 713 | (ref.) | 153 | (ref.) |
| **BMI** |  |  |  |  |  |  |
| < 18.5 (underweight) | 47 | 0.89 (0.41 - 1.93) | 31 | 1.09 (0.20 - 6.06) | 11 | 1.28 (0.26 - 6.35) |
| 18.5-24.9 (healthy) | 422 | (ref.) | 239 | (ref.) | 107 | (ref.) |
| 25-29.9 (overweight) | 743 | 0.72 (0.53 - 0.97)***** | 450 | 0.62 (0.31 - 1.25) | 161 | 0.95 (0.51 - 1.77) |
| 30-39.9 (obese) | 985 | 0.51 (0.38 - 0.69)******* | 624 | 0.38 (0.18 - 0.79)****** | 188 | 1.33 (0.70 - 2.52) |
| ≥ 40 (morbidly obese) | 374 | 0.40 (0.27 - 0.60)******* | 232 | 0.45 (0.17 - 1.22) | 55 | 0.96 (0.39 - 2.38) |
| **Non-HIV immune suppression** |  |  |  |  |  |  |
| Yes | 80 | 1.72 (1.02 - 2.90)***** | 40 | 2.62 (0.90 - 7.64) | 30 | 0.56 (0.22 - 1.44) |
| No | 2499 | (ref.) | 1540 | (ref.) | 495 | (ref.) |
| **Malignancy** |  |  |  |  |  |  |
| Yes | 105 | 2.40 (1.52 - 3.81)******* | 48 | 0.62 (0.13 - 3.01) | 39 | 0.44 (0.20 - 0.99)***** |
| No | 2474 | (ref.) | 1532 | (ref.) | 486 | (ref.) |
| **Pulmonary status** |  |  |  |  |  |  |
| No O2 | 644 | (ref.) | 405 | (ref.) | 93 | (ref.) |
| O2 < 4 L/min | 932 | 1.01 (0.75 - 1.36) | 564 | 1.03 (0.47 - 2.28) | 174 | 1.28 (0.64 - 2.55) |
| O2 ≥ 4 L/min | 719 | 1.46 (1.07 - 1.99)***** | 443 | 2.09 (0.91 - 4.76) | 182 | 1.09 (0.55 - 2.18) |
| Non-invasive vent./HFNC | 284 | 1.33 (0.87 - 2.02) | 168 | 4.46 (1.68 - 11.83)****** | 76 | 0.64 (0.25 - 1.61) |
| **Serum creatinine mg/dL** |  |  |  |  |  |  |
| < 1.1 | 1902 | (ref.) | 1211 | (ref.) | 347 | (ref.) |
| 1.1-1.5 | 400 | 1.04 (0.78 - 1.39) | 233 | 1.52 (0.78 - 2.95) | 87 | 0.56 (0.30 - 1.02) |
| > 1.5 | 274 | 1.70 (1.22 - 2.37)****** | 135 | 2.99 (1.41 - 6.38)****** | 90 | 0.27 (0.14 - 0.53)******* |
| **eGFR** |  |  |  |  |  |  |
| < 60 | 497 | 1.39 (1.07 - 1.81)***** | 250 | 2.39 (1.29 - 4.43)****** | 145 | 0.38 (0.23 - 0.64)******* |
| ≥ 60 | 2079 | (ref.) | 1329 | (ref.) | 379 | (ref.) |
| **CRP (mg/L)** |  |  |  |  |  |  |
| < 5 | 1633 | (ref.) | 1055 | (ref.) | 292 | (ref.) |
| 5-7.5 | 351 | 1.44 (1.09 - 1.89)****** | 193 | 1.43 (0.73 - 2.77) | 95 | 0.88 (0.49 - 1.58) |
| > 7.5 | 330 | 1.15 (0.86 - 1.53) | 193 | 1.18 (0.62 - 2.25) | 87 | 0.90 (0.49 - 1.66) |
| **IL-6 (ng/L)** |  |  |  |  |  |  |
| ≤ 5.8 | 1207 | (ref.) | 750 | (ref.) | 214 | (ref.) |
| > 5.8 | 1227 | 0.81 (0.65 - 1.02) | 756 | 2.04 (1.13 - 3.71)***** | 286 | 1.04 (0.62 - 1.74) |
| **D-dimer (mg/L)** |  |  |  |  |  |  |
| ≤ 0.93 | 1213 | (ref.) | 799 | (ref.) | 189 | (ref.) |
| > 0.93 | 1221 | 1.65 (1.32 - 2.05)******* | 707 | 0.99 (0.58 - 1.69) | 311 | 1.12 (0.69 - 1.81) |
| **Corticosteroid use** |  |  |  |  |  |  |
| Yes | 1756 | 1.32 (1.02 - 1.70)***** | 1051 | 0.63 (0.34 - 1.16) | 399 | 1.44 (0.81 - 2.57) |
| No | 823 | (ref.) | 529 | (ref.) | 126 | (ref.) |
| **Aspirin use** |  |  |  |  |  |  |
| Yes | 385 | 1.34 (1.02 - 1.75)***** | 202 | 1.43 (0.76 - 2.70) | 109 | 0.78 (0.46 - 1.33) |
| No | 2194 | (ref.) | 1378 | (ref.) | 416 | (ref.) |
| **Heparin use** |  |  |  |  |  |  |
| None | 815 | (ref.) | 478 | (ref.) | 158 | (ref.) |
| Prophylactic dose | 1515 | 0.95 (0.75 - 1.21) | 936 | 1.18 (0.65 - 2.11) | 320 | 1.02 (0.61 - 1.70) |
| Intermediate/therapeutic dose | 249 | 0.80 (0.54 - 1.18) | 166 | 0.70 (0.25 - 1.97) | 47 | 4.35 (1.47 - 12.89)****** |
| **Antirejection medication** |  |  |  |  |  |  |
| Yes | 101 | 2.59 (1.61 - 4.19)******* | 39 | 2.28 (0.79 - 6.57) | 44 | 0.43 (0.19 - 0.99)***** |
| No | 2478 | (ref.) | 1541 | (ref.) | 481 | (ref.) |
|  | | | | | | |
| * Significant p-values are denoted as follows next to parentheses: * p-value < 0.05, ** p-value < 0.01, *** p-value < 0.001  ^†^ Adjusted for age, sex, race/ethnicity, residence, geographical region, date of infection, baseline pulmonary status, Quanterix Ag, SARS-CoV-2 viral load, eGFR, CRP, IL-6, D-dimer, corticosteroid use, immunomodulator use, and remdesivir use | | | | | | |
| ^‡^ Adjusted for age, sex, race/ethnicity, residence, geographical region, date of infection, baseline pulmonary status, Quanterix Ag, SARS-CoV-2 viral load, eGFR, CRP, IL-6, D-dimer, D0 and D5 corticosteroid use, D0 and D5 immunomodulator use, D0 and D5 remdesivir use, and randomized treatment group  All variables tested in the lymphopenia analyses were also examined for severe lymphopenia. Only those that were significant (p-value < 0.05) were included in this table.  Ab=antibody, Ag=antigen, BMI=body mass index, HFNC=high flow nasal canula, NEWS=National Early Warning Score, eGFR=estimated glomerular filtration rate, CRP=C-reactive protein, IL-6=interleukin 6 | | | | | | |
|  | | | | | | |
|  | | | | | | |
|  | | | | | | |
|  | | | | | | |
|  | | | | | | |
|  | | | | | | |
|  | | | | | | |

## Table E13: COVID-19 Related Medications associated with Day 0 Lymphopenia, New (Day 5) Lymphopenia, or Resolved (Day 5) Lymphopenia

|  | **D0 Lymphopenia** | | **New Lymphopenia compared to No Lymphopenia** | | **Resolved Lymphopenia compared to Persistent Lymphopenia** | |
| --- | --- | --- | --- | --- | --- | --- |
|  | **N Pts.** | **OR (95% CI)^†^** | **N Pts.** | **OR (95% CI)^‡^** | **N Pts.** | **OR (95% CI)^‡^** |
| **Corticosteroids** |  |  |  |  |  |  |
| Baseline |  |  |  |  |  |  |
| Yes | 1756 | 1.31 (1.05 - 1.62)***** | 581 | 1.27 (0.65 - 2.49) | 869 | 1.33 (0.93 - 1.89) |
| No | 823 | (ref.) | 339 | (ref.) | 316 | (ref.) |
| Day 5 |  |  |  |  |  |  |
| Yes |  |  | 445 | 1.49 (0.79 - 2.81) | 719 | 0.64 (0.46 - 0.89)****** |
| No |  |  | 471 | (ref.) | 461 | (ref.) |
| Baseline or D5 use |  |  |  |  |  |  |
| Yes |  |  | 659 | 2.13 (0.97 - 4.71) | 968 | 1.14 (0.77 - 1.68) |
| No |  |  | 261 | (ref.) | 217 | (ref.) |
| **Immunomodulators** |  |  |  |  |  |  |
| Baseline |  |  |  |  |  |  |
| Yes | 168 | 1.26 (0.86 - 1.86) | 53 | 0.47 (0.14 - 1.55) | 97 | 1.40 (0.80 - 2.44) |
| No | 2411 | (ref.) | 867 | (ref.) | 1088 | (ref.) |
| Day 5 |  |  |  |  |  |  |
| Yes |  |  | 55 | 1.56 (0.54 - 4.46) | 84 | 0.77 (0.44 - 1.36) |
| No |  |  | 861 | (ref.) | 1096 | (ref.) |
| Baseline or D5 use |  |  |  |  |  |  |
| Yes |  |  | 76 | 0.84 (0.37 - 1.93) | 142 | 1.05 (0.67 - 1.65) |
| No |  |  | 844 | (ref.) | 1043 | (ref.) |
| **Remdesivir** |  |  |  |  |  |  |
| Baseline |  |  |  |  |  |  |
| Yes | 1574 | 0.92 (0.75 - 1.13) | 535 | 1.14 (0.62 - 2.11) | 748 | 0.74 (0.53 - 1.03) |
| No | 1005 | (ref.) | 385 | (ref.) | 437 | (ref.) |
| Day 5 |  |  |  |  |  |  |
| Yes |  |  | 341 | 1.22 (0.68 - 2.18) | 456 | 0.94 (0.69 - 1.29) |
| No |  |  | 579 | (ref.) | 729 | (ref.) |
| Baseline or D5 use |  |  |  |  |  |  |
| Yes |  |  | 760 | 0.77 (0.36 - 1.65) | 994 | 0.71 (0.48 - 1.07) |
| No |  |  | 160 | (ref.) | 191 | (ref.) |
| **Heparin** |  |  |  |  |  |  |
| Baseline |  |  |  |  |  |  |
| None | 815 | (ref.) | 288 | (ref.) | 348 | (ref.) |
| Prophylactic dose | 1515 | 1.06 (0.86 - 1.32) | 526 | 1.25 (0.66 - 2.36) | 730 | 1.07 (0.77 - 1.47) |
| Inter./therapeutic dose | 249 | 0.77 (0.55 - 1.08) | 106 | 0.70 (0.25 - 1.98) | 107 | 1.53 (0.89 - 2.64) |
| Day 5 |  |  |  |  |  |  |
| None |  |  | 515 | (ref.) | 604 | (ref.) |
| Prophylactic dose |  |  | 328 | 0.76 (0.39 - 1.46) | 468 | 1.10 (0.80 - 1.53) |
| Inter./therapeutic dose |  |  | 77 | 0.28 (0.09 - 0.87)***** | 113 | 1.13 (0.67 - 1.90) |
| Baseline or D5 use |  |  |  |  |  |  |
| None |  |  | 229 | (ref.) | 261 | (ref.) |
| Prophylactic dose |  |  | 551 | 0.96 (0.48 - 1.94) | 753 | 1.05 (0.73 - 1.50) |
| Inter./therapeutic dose |  |  | 140 | 0.53 (0.20 - 1.42) | 171 | 1.12 (0.69 - 1.82) |
| **Randomized treatment** |  |  |  |  |  |  |
| Active | 1452 | 0.92 (0.77 - 1.10) | 503 | 0.76 (0.45 - 1.27) | 657 | 1.18 (0.89 - 1.55) |
| Placebo | 1127 | (ref.) | 417 | (ref.) | 528 | (ref.) |
| * Significant p-values are denoted as follows next to parentheses: * p-value < 0.05, ** p-value < 0.01, *** p-value < 0.001  ^†^ Adjusted for age, sex, race/ethnicity, residence, geographical region, date of infection, baseline pulmonary status, Quanterix Ag, SARS-CoV-2 viral load, eGFR, CRP, IL-6, D-dimer, corticosteroid use, immunomodulator use, and remdesivir use | | | | | | |
| ^‡^ Adjusted for age, sex, race/ethnicity, residence, geographical region, date of infection, baseline pulmonary status, Quanterix Ag, SARS-CoV-2 viral load, eGFR, CRP, IL-6, D-dimer, D0 and D5 corticosteroid use, D0 and D5 immunomodulator use, D0 and D5 remdesivir use, and randomized treatment group  Corticosteroid, immunomodulator, and heparin use was assessed for the 24 hours prior to randomization and the 24 hours prior to the day 5 visit. Baseline remdesivir use was defined by any dose received prior to randomization. Day 5 remdesivir use was defined as a dose received on day 4 or day 5. | | | | | | |

## Table E14: COVID-19 Related Medications associated with Day 0 Severe Lymphopenia, New (Day 5) Severe Lymphopenia, or Resolved (Day 5) Severe Lymphopenia

|  | **D0 Lymphopenia** | | **New Severe Lymphopenia compared to No Severe Lymphopenia** | | **Resolved Severe Lymphopenia compared to Persistent Severe Lymphopenia** | |
| --- | --- | --- | --- | --- | --- | --- |
|  | **N Pts.** | **OR (95% CI)^†^** | **N Pts.** | **OR (95% CI)^‡^** | **N Pts.** | **OR (95% CI)^‡^** |
| **Corticosteroids** |  |  |  |  |  |  |
| Baseline |  |  |  |  |  |  |
| Yes | 1756 | 1.32 (1.02 - 1.70)***** | 1051 | 0.63 (0.34 - 1.16) | 399 | 1.44 (0.81 - 2.57) |
| No | 823 | (ref.) | 529 | (ref.) | 126 | (ref.) |
| Day 5 |  |  |  |  |  |  |
| Yes |  |  | 819 | 1.31 (0.72 - 2.39) | 345 | 0.85 (0.50 - 1.44) |
| No |  |  | 756 | (ref.) | 176 | (ref.) |
| Baseline or D5 use |  |  |  |  |  |  |
| Yes |  |  | 1184 | 0.87 (0.43 - 1.73) | 443 | 1.64 (0.84 - 3.22) |
| No |  |  | 396 | (ref.) | 82 | (ref.) |
| **Immunomodulators** |  |  |  |  |  |  |
| Baseline |  |  |  |  |  |  |
| Yes | 168 | 1.36 (0.91 - 2.03) | 100 | 0.34 (0.11 - 1.06) | 50 | 1.68 (0.71 - 3.98) |
| No | 2411 | (ref.) | 1480 | (ref.) | 475 | (ref.) |
| Day 5 |  |  |  |  |  |  |
| Yes |  |  | 99 | 2.78 (1.14 - 6.79)***** | 40 | 0.46 (0.19 - 1.11) |
| No |  |  | 1476 | (ref.) | 481 | (ref.) |
| Baseline or D5 use |  |  |  |  |  |  |
| Yes |  |  | 147 | 0.98 (0.45 - 2.15) | 71 | 0.82 (0.42 - 1.60) |
| No |  |  | 1433 | (ref.) | 454 | (ref.) |
| **Remdesivir** |  |  |  |  |  |  |
| Baseline |  |  |  |  |  |  |
| Yes | 1574 | 1.07 (0.85 - 1.36) | 938 | 1.10 (0.62 - 1.95) | 345 | 1.05 (0.59 - 1.85) |
| No | 1005 | (ref.) | 642 | (ref.) | 180 | (ref.) |
| Day 5 |  |  |  |  |  |  |
| Yes |  |  | 582 | 1.01 (0.58 - 1.75) | 215 | 1.15 (0.69 - 1.89) |
| No |  |  | 998 | (ref.) | 310 | (ref.) |
| Baseline or D5 use |  |  |  |  |  |  |
| Yes |  |  | 1291 | 1.47 (0.67 - 3.20) | 463 | 1.20 (0.58 - 2.49) |
| No |  |  | 289 | (ref.) | 62 | (ref.) |
| **Heparin** |  |  |  |  |  |  |
| Baseline |  |  |  |  |  |  |
| None | 815 | (ref.) | 478 | (ref.) | 158 | (ref.) |
| Prophylactic dose | 1515 | 0.95 (0.75 - 1.21) | 936 | 1.18 (0.65 - 2.11) | 320 | 1.02 (0.61 - 1.70) |
| Intermediate/therapeutic dose | 249 | 0.80 (0.54 - 1.18) | 166 | 0.70 (0.25 - 1.97) | 47 | 4.35 (1.47 - 12.89)****** |
| Day 5 |  |  |  |  |  |  |
| None |  |  | 874 | (ref.) | 245 | (ref.) |
| Prophylactic dose |  |  | 579 | 1.32 (0.73 - 2.40) | 217 | 1.31 (0.78 - 2.19) |
| Intermediate/therapeutic dose |  |  | 127 | 0.89 (0.34 - 2.36) | 63 | 2.27 (1.02 - 5.06)***** |
| Baseline or D5 use |  |  |  |  |  |  |
| None |  |  | 377 | (ref.) | 113 | (ref.) |
| Prophylactic dose |  |  | 977 | 1.40 (0.71 - 2.76) | 327 | 1.26 (0.71 - 2.24) |
| Intermediate/therapeutic dose |  |  | 226 | 0.66 (0.25 - 1.77) | 85 | 2.74 (1.23 - 6.10)***** |
| **Randomized treatment** |  |  |  |  |  |  |
| Active | 1452 | 0.83 (0.68 - 1.02) | 878 | 0.71 (0.43 - 1.17) | 282 | 1.16 (0.74 - 1.82) |
| Placebo | 1127 | (ref.) | 702 | (ref.) | 243 | (ref.) |
| * Significant p-values are denoted as follows next to parentheses: * p-value < 0.05, ** p-value < 0.01, *** p-value < 0.001  ^†^ Adjusted for age, sex, race/ethnicity, residence, geographical region, date of infection, baseline pulmonary status, Quanterix Ag, SARS-CoV-2 viral load, eGFR, CRP, IL-6, D-dimer, corticosteroid use, immunomodulator use, and remdesivir use | | | | | | |
| ^‡^ Adjusted for age, sex, race/ethnicity, residence, geographical region, date of infection, baseline pulmonary status, Quanterix Ag, SARS-CoV-2 viral load, eGFR, CRP, IL-6, D-dimer, D0 and D5 corticosteroid use, D0 and D5 immunomodulator use, D0 and D5 remdesivir use, and randomized treatment group  Corticosteroid, immunomodulator, and heparin use was assessed for the 24 hours prior to randomization and the 24 hours prior to the day 5 visit. Baseline remdesivir use was defined by any dose received prior to randomization. Day 5 remdesivir use was defined as a dose received on day 4 or day 5. | | | | | | |

## Figure E8: Association of COVID-19 Related Medications with New lymphopenia when compared to no lymphopenia and resolved lymphopenia when compared to persistent lymphopenia

**
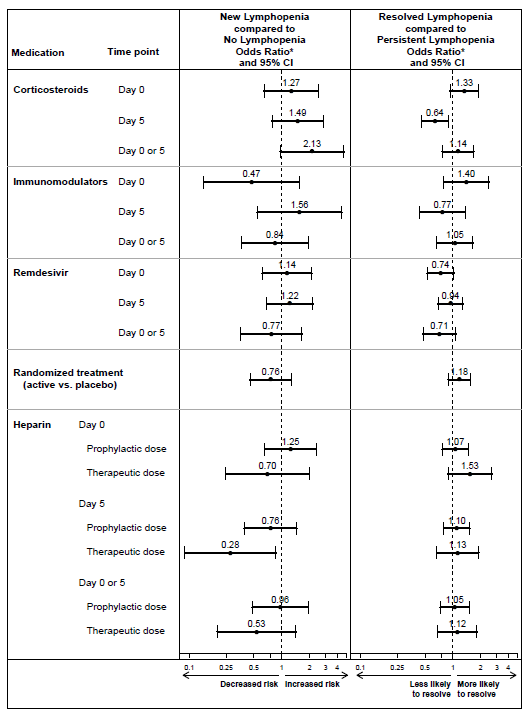
**

*Adjusted for age, sex, race/ethnicity, residence, geographical region, date of infection, baseline pulmonary status, Quanterix Ag, SARS-CoV-2 viral load, eGFR, CRP, IL-6, D-dimer, D0 and D5 corticosteroid use, D0 and D5 immunomodulator use, D0 and D5 remdesivir use, and randomized treatment group

## Figure E9: Severe Lymphopenia association with COVID treatments


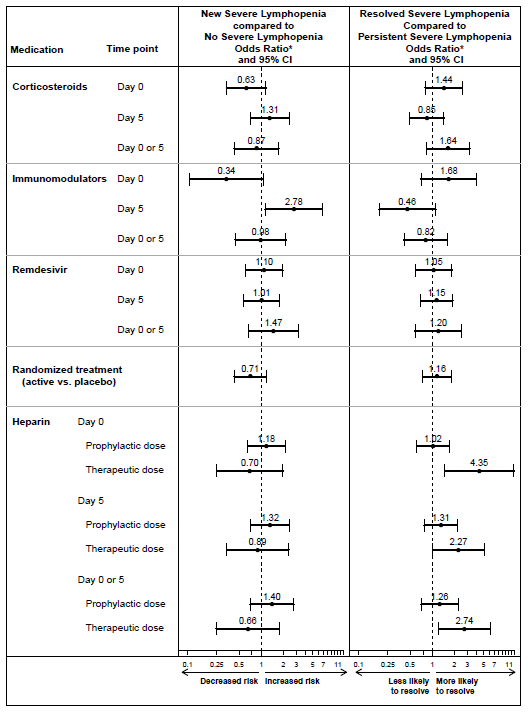


*Adjusted for age, sex, race/ethnicity, residence, geographical region, date of infection, baseline pulmonary status, Quanterix Ag, SARS-CoV-2 viral load, eGFR, CRP, IL-6, D-dimer, D0 and D5 corticosteroid use, D0 and D5 immunomodulator use, D0 and D5 remdesivir use, and randomized treatment group

## Figure E10: Longitudinal biomarker measurements by severe lymphopenia trajectory groups


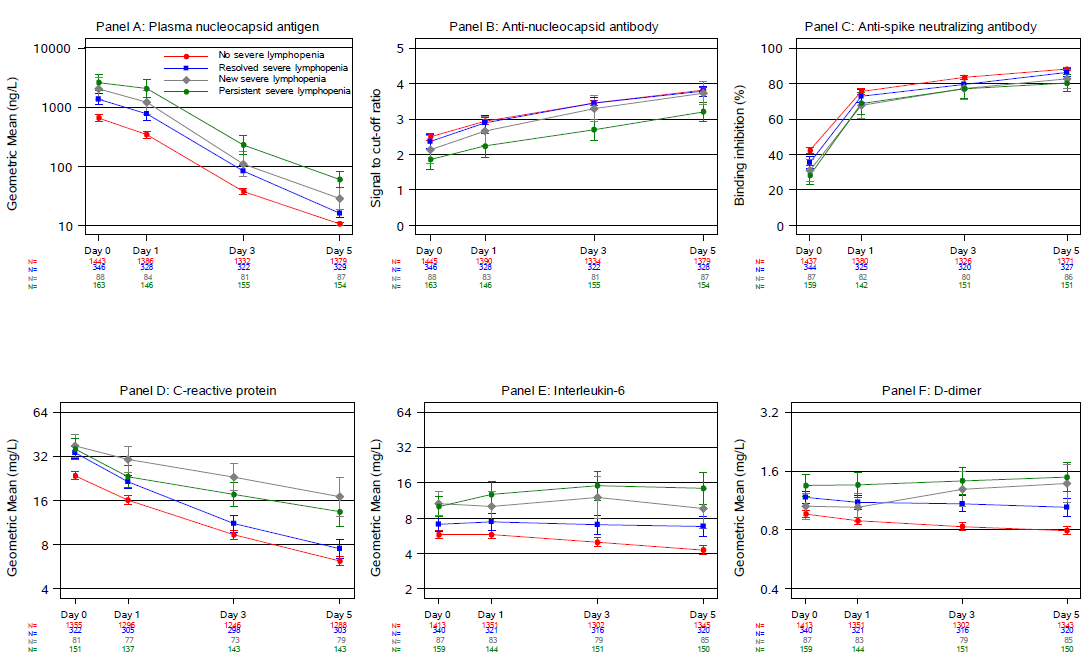


Biomarker measurements at Days 0, 1, 3, and 5 by severe lymphopenia trajectory group. Plasma nucleocapsid antigen (Panel A), C-reactive protein (Panel D), interleukin-6 (Panel E), and D-dimer (Panel F) are non-normally distributed and are summarized by geometric means. Anti-nucleocapsid antibody (Panel B) and anti-spike neutralizing antibody (Panel C) are displayed as means on the original scale.

1. Rogers AJ, Wentworth D, Phillips A, Shaw-Saliba K, Dewar RL, Aggarwal NR, et al. The Association of Baseline Plasma SARS-CoV-2 Nucleocapsid Antigen Level and Outcomes in Patients Hospitalized With COVID-19. Ann Intern Med 2022;175(10):1401-1410.

2. Aggarwal NR, Nordwall J, Braun DL, Chung L, Coslet J, Der T, et al. Viral and Host Factors Are Associated With Mortality in Hospitalized Patients With COVID-19. Clin Infect Dis 2024.
